# Hepatic Steatosis Aggravates Vascular Calcification via Extracellular Vesicle‐Mediated Osteochondrogenic Switch of Vascular Smooth Muscle Cells

**DOI:** 10.1002/advs.202408660

**Published:** 2024-12-16

**Authors:** Zhao‐Lin Zeng, Zhi‐Bo Zhao, Qing Yuan, Shi‐Qi Yang, Zhen‐Xing Wang, Zuo Wang, Shi‐Yu Zeng, An‐Qi Li, Qian Chen, Guo‐Qiang Zhu, Xin‐Hua Xiao, Guang‐Hua Luo, Hai‐Yan Luo, Jiao‐Yang Li, Xu‐Yu Zu, Hui Xie, Jiang‐Hua Liu

**Affiliations:** ^1^ Department of Metabolism and Endocrinology The First Affiliated Hospital, Hengyang Medical School University of South China Hengyang Hunan 421001 P. R. China; ^2^ Department of Cardiovascular Medicine The First Affiliated Hospital of Chongqing Medical University Chongqing 400016 P. R. China; ^3^ Diabetes Clinical Medical Research Center of Hunan Provincial Hengyang Hunan 421001 P. R. China; ^4^ Department of Clinical Laboratory Medicine The First Affiliated Hospital Hengyang Medical School University of South China Hengyang Hunan 421001 P. R. China; ^5^ Institute of Cardiovascular Disease Key Lab for Arteriosclerology of Hunan Province Hengyang Medical School University of South China Hengyang Hunan 421001 P. R. China; ^6^ Department of Orthopedics Movement System Injury and Repair Research Center National Clinical Research Center for Geriatric Disorders Hunan Key Laboratory of Angmedicine Xiangya Hospital Central South University Changsha Hunan 410008 P. R. China; ^7^ Department of Radiology The First Affiliated Hospital Hengyang Medical School University of South China Hengyang Hunan 421001 P. R. China; ^8^ Department of Gastroenterology The First Affiliated Hospital Hengyang Medical School University of South China Hengyang Hunan 421001 P. R. China; ^9^ Department of Occupational and Environmental Health School of Public Health Wuhan University Wuhan 430071 P. R. China

**Keywords:** atherosclerosis, extracellular vesicles, Lgals3bp, MAFLD, vascular calcification

## Abstract

The global incidence of metabolic dysfunction‐associated fatty liver disease (MAFLD) has risen sharply. This condition is strongly associated with the risk of cardiovascular disease (CVD), but how MAFLD affects the development and progression of CVD, particularly concerning vascular calcification, remains unclear. Herein, extracellular vesicles (EVs) are identified from steatotic hepatocytes as a trigger that accelerated the progression of both vascular intimal and medial calcification. Steatotic hepatocytes are found to release more EVs, which are able to reach the vascular tissue, be taken up by vascular smooth muscle cells (VSMCs), and promote their osteogenic differentiation. Within these toxic vesicles, a protein cargo is identified called lectin galactoside‐binding soluble 3 binding protein (Lgals3bp) that acted as a potent inducer of osteochondrogenic transformation in VSMCs. Both the inhibition of EV release and the liver‐specific knockdown of Lgals3bp profoundly attenuated vascular calcification. This work partially explains the reason for the high incidence of vascular calcification in MAFLD and unveils a novel mechanism that may be used to prevent or treat cardiovascular complications in patients with MAFLD.

## Graphical abstract

Steatotic hepatocytes‐derived extracellular vesicles (SHep‐EVs) in the liver of mice with metabolic dysfunction‐associated fatty liver disease (MAFLD) can be transferred to vascular smooth muscle cells (VSMCs), exacerbating atherosclerosis and calcification by delivering Lgals3bp.

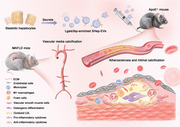

## 1 Introduction

Metabolic dysfunction‐associated fatty liver disease (MAFLD), previously known as non‐alcoholic fatty liver disease (NAFLD),^[^ ^1^ ^]^ has had a recent surge in prevalence. Globally, MAFLD affects 25% of the population, including 55.5% of individuals with type 2 diabetes mellitus (T2DM).^[^ ^2^ ^]^ In China, its prevalence has reached a staggering 32.9%, imposing a substantial health burden on patients.^[^ ^3^ ^]^ MAFLD not only affects the liver itself but also contributes to extrahepatic comorbidities,^[^ ^4^ ^]^ including cardiovascular diseases (CVDs),^[^ ^5^ ^]^ osteoporosis,^[^ ^6^ ^]^ chronic kidney disease,^[^ ^7^ ^]^ and depressive disorder.^[^ ^8^ ^]^ Increasing evidence has shown that extrahepatic complications have a far greater impact on patients than on the liver itself. It has been conclusively demonstrated that CVDs are the leading cause of death in MAFLD patients, followed by liver disease.^[^ ^9^ ^]^

Vascular calcification can be divided into at least five categories according to the location of the lesion: intimal calcification, medial calcification, adventitial calcification, valvular calcification, and calciphylaxis.^[^ ^10^ ^]^ Intimal calcification is common in atherosclerotic lesions and is considered one of the hallmarks of the progression of atherosclerosis. Major risk factors include hyperlipidemia and inflammation, and intimal calcification is associated with the risk of plaque rupture.^[^ ^11^ ^]^ Medial calcification is common in patients with metabolic imbalances such as diabetes and chronic kidney disease (CKD) and is associated with a loss of vascular elasticity and arterial dissection. The osteogenic differentiation of VSMCs is currently considered to be the main mechanism through which medial calcification occurs.^[^ ^12^ ^]^ Vascular calcification is strongly correlated with CVD‐related morbidity and mortality. However, there is still a lack of effective treatment therapies due to the poorly defined molecular mechanisms governing the calcification process in the vessel wall.^[^ ^10^, ^13^ ^]^ Recent studies have shed light on the fact that there is a noticeable increase in the degree of vascular calcification among individuals diagnosed with MAFLD.^[^ ^10^, ^14^ ^]^

Previous studies exploring the effects of MAFLD on vascular calcification were mainly limited to the clinical level, and there is still a lack of relevant animal experiments and mechanistic studies. MAFLD is often associated with metabolic imbalance and chronic inflammation.^[^ ^15^ ^]^ Therefore, it is of great clinical significance to explore the specific mechanisms underlying MAFLD‐induced vascular intimal and medial calcification onset and progression and to then out effective approaches to preventing and controlling this form of pathology.

Extracellular vesicles (EVs) usually include vesicles smaller than 200 nm in diameter, including both exosomes and microvesicles,^[^ ^16^ ^]^ that play crucial roles in nucleic acid and protein transport, antigen presentation, regulation of the cellular micro‐environment, and inter‐organ cross‐talk.^[^ ^17^ ^]^ Interdisciplinary studies have increasingly begun to focus on the role of EVs in calcified vascular lesions. Ultrastructural analyses have confirmed the enrichment of EVs in calcified lesions present in human aortic valves and arteries.^[^ ^18^ ^]^ Previous studies have reported that steatotic hepatocyte‐derived EVs (SHep‐EVs) can promote atherosclerosis by exacerbating endothelial inflammatory responses,^[^ ^19^ ^]^ while intimal calcification, which often appears with advanced atherosclerosis, is a specific manifestation of soft tissue calcification that arises under conditions such as chronic inflammation.^[^ ^20^ ^]^ We thus speculated that SHep‐EVs may promote vascular calcification by mediating crosstalk between the liver and vasculature.

In this study, we uncovered the role of the SHep‐EV as messengers that can promote vascular calcification by transferring specific proteins to vascular smooth muscle cells (VSMCs). We isolated EVs from both normal and steatotic hepatocytes, characterized these EVs, and found that steatotic hepatocytes secrete more EVs than healthy hepatocytes. In vitro, SHep‐EV promoted the osteogenic differentiation of VSMCs. In vivo, we confirmed that MAFLD was able to promote vitamin D (VitD) overload‐induced vascular calcification (a widely used and reproducible model of medial calcification),^[^ ^21^ ^]^ uremia‐related vascular medial calcification, and to facilitate atherosclerotic vascular intimal calcification in ApoE^−/−^ mice. The liver‐specific blockade of EV secretion was sufficient to alleviate such MAFLD‐associated atherosclerosis and calcification.

Through proteomic analyses, we additionally found that SHep‐EV contained elevated levels of the lectin galactoside‐binding soluble 3binding protein (Lgals3bp), which promotes VSMC osteogenic differentiation. Finally, we confirmed that hepatocyte‐specific knockout of Lgals3bp significantly inhibited MAFLD‐associated atherosclerosis and calcification. In conclusion, our work suggests that hepatocyte‐derived EVs in fatty liver tissue can be transferred into the vascular tissue and aggravate atherosclerosis and calcification by transferring Lgals3bp, indicating that this mechanism may represent an important therapeutic target for MAFLD and its extrahepatic complications.

## 2 Results

### 2.1 MAFLD is Positively Associated with Arterial Calcification

To determine whether MAFLD affects the process of vascular calcification, we first analyzed the clinical correlation between MAFLD and vascular calcification using data from our previous registered clinical research project (NCT04889053), with all enrolled patients having T2DM. A total of 583 subjects met the inclusion criteria for that study (inclusion and exclusion criteria are shown in the Methods section). For the current cross sectional study, we excluded 63 subjects with missing liver fat content‐related information (*n* = 26), viral hepatitis (*n* = 9), or CKD (*n* = 28), while 520 participants (321 MAFLD patients and 199 Non‐MAFLD patient) were included in the final analysis (Figure S1, Supporting Information).

The mean age (standard deviation [SD]) and mean body mass index (BMI) (interquartile range) of 520 these participants were 54.6 years (9.8) and 23.79 kg m^−2^ (21.54–26.12), respectively, and 59.8% of these participants were male. The prevalence of CAC scores > 0 was 55.8%, with the majority of these subjects exhibiting coronary artery calcium scores (CACS) between 1 to 100 (83.9%). The characteristics of these study subjects are shown in Table S1 (Supporting Information). There were no significant differences for most baseline comparisons between the non‐MAFLD and MAFLD groups, but BMI and 25(OH)D levels have significant differences between the two groups. In addition, relative to the non‐MAFLD group, the MAFLD group exhibited higher triglyceride (TG), glucose (Glu), alanine transaminase (ALT), aspartate transaminase (AST), and serum calcium levels, as well as lower levels of parathyroid hormone (PTH). Of the study subjects, Non‐MAFLD are199, and MFALD are 321 (61.7%), this is consistent with the previously reported prevalence of fatty liver in T2DM of 65.04%.^[^ ^22^ ^]^ The odds of patients with fatty liver having coronary artery calcification (CACS > 0) were significantly higher than that of patients without fatty liver (66.0 vs. 39.2%) (Table S1, Supporting Information). Linear regression analysis demonstrated that among the various factors that may affect vascular calcification (including gender, age, BMI, diabetes duration, Glu, HbA1c, SBP, TC, fatty liver), fatty liver, age, and the duration of diabetes are positively correlated with vascular calcification (Table S2, Supporting Information). Moreover, higher liver fat content was associated with a greater risk of coronary artery calcification after adjusting for confounding factors (adjusted odds ratio [OR], 1.41; 95% confidence interval [CI], 1.28‐1.56; *p *< 0.001) (Table S3, Supporting Information).

**Figure 1:**
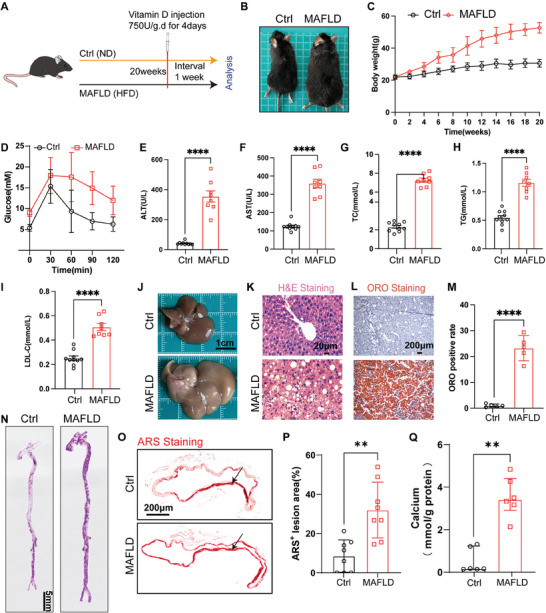
MAFLD is positively associated with arterial calcification. A) Schematic diagram of the animal experimental approach; B) Mouse body size comparison; C) Body weight (*n* = 10); D) Glucose tolerance analysis (*n* = 8); E,F) Liver function analysis (*n* = 8 or 10); G–I) Blood lipid analysis (*n* = 8 or 10); J) Comparison of liver morphology, scale bar = 1 cm; K) Representative images of liver tissue sections following hematoxylin and eosin (H&E) staining, scale bar = 20 µm; L,M) Oil red O (ORO) staining and corresponding analyses for liver tissue sections (*n* = 5), scale bar = 200 µm; N) Alizarin red S (ARS) staining of the whole aorta (purple) (scale bar = 5 mm); O,P) Representative images of ARS staining of abdominal aorta sections and corresponding statistical analyses of the percentage of ARS‐positive area to the total vascular area (*n* = 8), calcified vascular tissue is dark red, scale bar = 200 µm; Q) Aortic calcium content was analyzed and corrected for total protein (*n* = 6). Data are presented as means ± SD (Figure 1E–K,O,R) or median (interquartile range) (Figure 1Q), *p* values were determined by unpaired two‐tailed Student's t‐test (Figure 1E–I,M,P), and nonparametric tests (Mann‐Whitney U test) (Figure 1Q); **p < 0.01, ****p < 0.0001. Ctrl: Regular diet group; MAFLD: high‐fat diet group.

We additionally observed the effects of MAFLD on vascular calcification in animals. High‐dose VitD is often used to construct a vascular medial calcification model by increasing serum calcium and phosphate levels and inducing the osteogenic differentiation of VSMCs.^[^ ^21^, ^23^ ^]^ To clarify the effect of MAFLD on vascular calcification, we established a MAFLD‐related vascular calcification model by combining a high‐fat diet (HFD, R&D 12492) with the intraperitoneal injection of VitD (**Figure** **1**A). After 20 weeks of HFD feeding, the mice in the MAFLD group exhibited significant weight gain and obesity (Figure 1B,C), together with impaired glucose tolerance (Figure 1D). They also presented with significant increases in the levels of the liver function indicators ALT and AST (Figure 1E,F), their blood total cholesterol (TC), glycerides (TG), and low‐density lipoprotein cholesterol (LDL‐c) levels were all elevated (Figure 1G–I). Macroscopic observation showed that the liver of MAFLD mice was enlarged and whitened (Figure 1J). Hematoxylin and eosin (H&E) staining of liver tissue sections from MAFLD mice revealed the formation of many vacuoles (Figure 1K). Oil red O (ORO) staining highlighted the formation of many orange‐red lipid droplets (Figure 1L,M). These data collectively indicated that the MAFLD model was successfully constructed.

The degree of calcification in the murine aorta was then evaluated. Alizarin red S (ARS) staining of the whole aorta (Figure 1N) and the abdominal aorta sections (Figure 1O,P) revealed MAFLD significantly increased the ARS positivity rate. In addition, MAFLD also increased aortic calcium content (Figure 1Q). Moreover, we also observed the effect of MAFLD on vascular calcification in mouse models of natural aging and CKD. After 3 months of HFD feeding (R&D12492) in 15‐month‐old C57 mice, the expression of the osteogenesis marker BMP2 in the aortic was significantly higher than that in the regular diet group, with a corresponding significant increase in aortic calcium content (Figure S2A,B, Supporting Information).

DBA/2 is a calcification‐prone mouse strain, and an increase in the severity of soft tissue calcification was also observed in DBA/2 mice with age. Moreover, the severity of calcification was higher in females relative to males.^[^ ^24^ ^]^ Dietary adenine administration can induce a stable and similar degree of chronic uremia‐related vascular calcification in DBA/2 mice.^[^ ^25^ ^]^ By adding 60% fat to simulate the pathogenesis of MAFLD, we found that HFD (R&D 12492) exacerbated adenine‐ and high‐phosphate‐induced vascular calcification in these animals (Figure S3, Supporting Information).

These clinical and animal model results confirm that MAFLD has a vascular calcification‐promoting effect. Overall, these collective results support the hypothesis that MAFLD accelerates vascular calcification.

### 2.2 Identification and Characterization of Hepatocyte‐Derived EVs

EVs are important mediators of interactions between organs.^[^ ^26^ ^]^ Many previous studies have reported the involvement of EVs in vascular calcification.^[^ ^21^, ^27^ ^]^ Therefore, we speculated that SHep‐EV may mediate the enhanced vascular calcification that occurs in MAFLD. Hepatocyte steatosis during MAFLD development was first simulated by treating hepatocytes with free fatty acids (FFAs) (oleic acid: palmitic acid V/V = 2:1) for 24 h.^[^ ^28^ ^]^ Cell viability assays showed that FFAs at concentrations above 200 µm inhibited hepatocyte viability (10–20%) (Figure S4A, Supporting Information). ORO staining showed that FFAs increased hepatocyte lipid loading in a concentration‐dependent manner (100–600 µm) (Figure S4B,C, Supporting Information). Compared with the use of 100 µm FFAs, the lipid accumulation of AML12 hepatocytes treated with 500 µm FFA was significantly increased, but there was no significant difference in the effect on the cell viability, and a further increase in the concentration of FFAs did not significantly increase hepatocyte lipid loading but further inhibited hepatocyte activity. Therefore, a 500 µm FFA concentration was chosen as the working concentration in this study.

**Figure 2:**
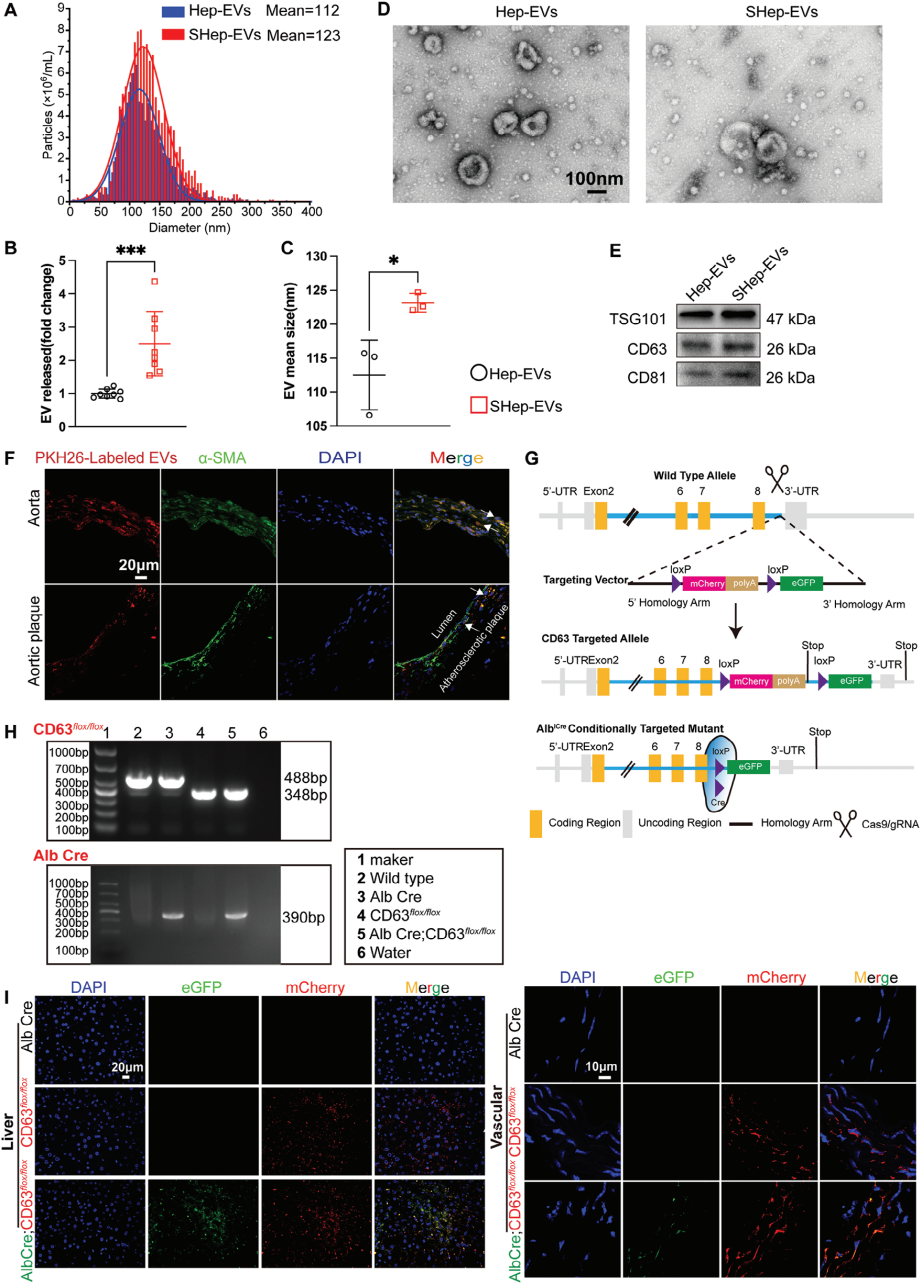
Identification of hepatocyte‐derived EVs and in vivo tracer analysis. A) Nanoparticle tracking analysis (NTA) analysis of EVs obtained by ultracentrifugation of medium from normal or steatotic AML‐12 mouse hepatocytes (*n* = 3); B) Analysis of the concentrations of Hep‐EVs and SHep‐EVs obtained by isolation from the same inoculum density and the same volume of cell culture medium using the bicinchoninic acid (BCA) method (*n* = 8); C) Particle size statistics for EVs (*n* = 3); D) Transmission electron microscope‐based detection of EVs, scale bar = 100 nm; E) Western blot analysis of the exosomal markers TSG101, CD63, and CD81. F,G) Fluorescence imaging of aortic (top) and aortic sinus (bottom) sections in ApoE^−/–^ mice after 24 weeks of weekly 75 µg PKH‐26‐labelled SHep‐EV injections via the tail vein, scale bar = 20 µm. G) Schematic representation of the construction of Alb Cre; Cd63em (loxp‐mCherry‐loxp‐eGFP)3 mice, which were generated by crossing Cd63em (loxp‐mCherry‐loxp‐eGFP)3 mice (with exosomes that express red fluorescence) with transgenic mice expressing Cre recombinase controlled by an Alb promoter. Cd63em(loxp‐mCherry‐loxp‐eGFP)3 mice, whose eGFP can be transcribed in hepatocytes due to Cre recombinase, allowing the exosomes to express green fluorescence; H) Genotypic identification results; I) Analysis of eGFP (green) and mCherry (red) fluorescence in the liver and vasculature, with EVs distributed as dots around the cell nucleus (in the cytoplasm) (*n* = 3), scale bar = 20 µm (Liver), or 10 µm (vasculature). Data are presented as means ± SD, and *p* values were determined using unpaired two‐tailed Student's t‐tests; *p < 0.05, *** *p* < 0.001.

EVs were isolated from the culture media of AML‐12 cells in normal medium (Hep‐EVs) or following treatment with FFAs (SHep‐EVs). Nanoparticle tracking analysis (NTA) revealed that Hep‐EVs and the SHep‐EV group had mean diameters of 112 ± 5.1 and 123 ± 1.4 nm, respectively, and there were nearly three times as many SHep‐EVs as Hep‐EVs (**Figure** **2**A–C). This finding is consistent with previous reports.^[^ ^29^ ^]^ Transmission electron microscopy (TEM) revealed that Hep‐EVs and SHep‐EVs exhibited cup‐like morphology (Figure 2D). EVs expressed high levels of the exosomal markers CD63, CD81, and TSG101 (Figure 2E). The above results suggested that we had isolated exosome‐enriched EVs.

Next, we performed in vivo experiments to directly test whether EVs released from hepatocytes could be transported to the vascular tissue and play a role in arterial calcification. We labeled hepatocytes‐derived EVs with the near‐infrared dye DiR and injected them into wild‐type mice via the tail vein. The results showed that hepatocyte‐derived EVs were mainly enriched in the liver, and also enriched to some extent in the heart, vascular, spleen, lung, kidney, and bone, with the degree of enrichment in the liver having further increased over time (6 to 24 h) (Figure S5, Supporting Information).

To further clarify whether hepatocyte‐derived EVs enter the vascular tissue and participate in the vascular calcification process, we injected PKH‐26‐labeled SHep‐EV into ApoE^−/−^ mice via the tail vein (75 µg per mouse, once per week), and aortic sections were taken for immunofluorescence analysis after 24 weeks. The results showed that PKH‐26‐labeled EVs can reside in vessel walls and atherosclerotic plaques (Figure 2F). In addition to examining the vascular tissue distribution of the SHep‐EV group in an atherosclerosis model (ApoE^−/‐^ mice), we used a Hep‐EV tracer mouse to examine the tissue distribution of the EVs. This was achieved using Cd63em(loxp‐mCherry‐loxp‐eGFP)3 mice crossed with transgenic mice expressing liver‐specific Cre recombinase under the control of the Alb promoter to generate Alb Cre; Cd63em(loxp‐mCherry‐loxp‐eGFP)3 mice (Figure 2G,H), in which eGFP could be transcribed in hepatocytes due to the deletion of the floxed mCherry by Cre recombinase, as detailed in our previous publication.^[^ ^21b^ ^]^ The mCherry and eGFP signal levels in the vascular sections of mice in different groups were assessed. As shown in Figure 2I, abundant mCherry red fluorescence was observed in the liver and vascular tissues of Cd63em(loxp‐mCherry‐loxp‐eGFP)3 and Alb Cre; Cd63em(loxp‐mCherry‐loxp‐eGFP)3 mice. However, abundant eGFP green signals could only be observed in Alb Cre; Cd63em(loxp‐mCherry‐loxp‐eGFP)3 mice in the perinuclear region of hepatocytes or the vascular tissues, suggesting that most of them are EVs released from hepatocytes that can enter into the vascular tissues. It is worth noting that the residence of Hep‐EVs in blood vessels was not tissue‐specific; in other words, Hep‐EVs were also able to reach other tissues, such as the kidneys and femurs (Figure S6, Supporting Information). Together, these findings indicate that EVs derived from hepatocytes can enter the vasculature and may modulate the osteogenic differentiation of VSMCs and participate in vascular calcification.

### 2.3 SHep‐EVs Accelerate Intimal Calcification in ApoE^−/−^ mice

**Figure 3:**
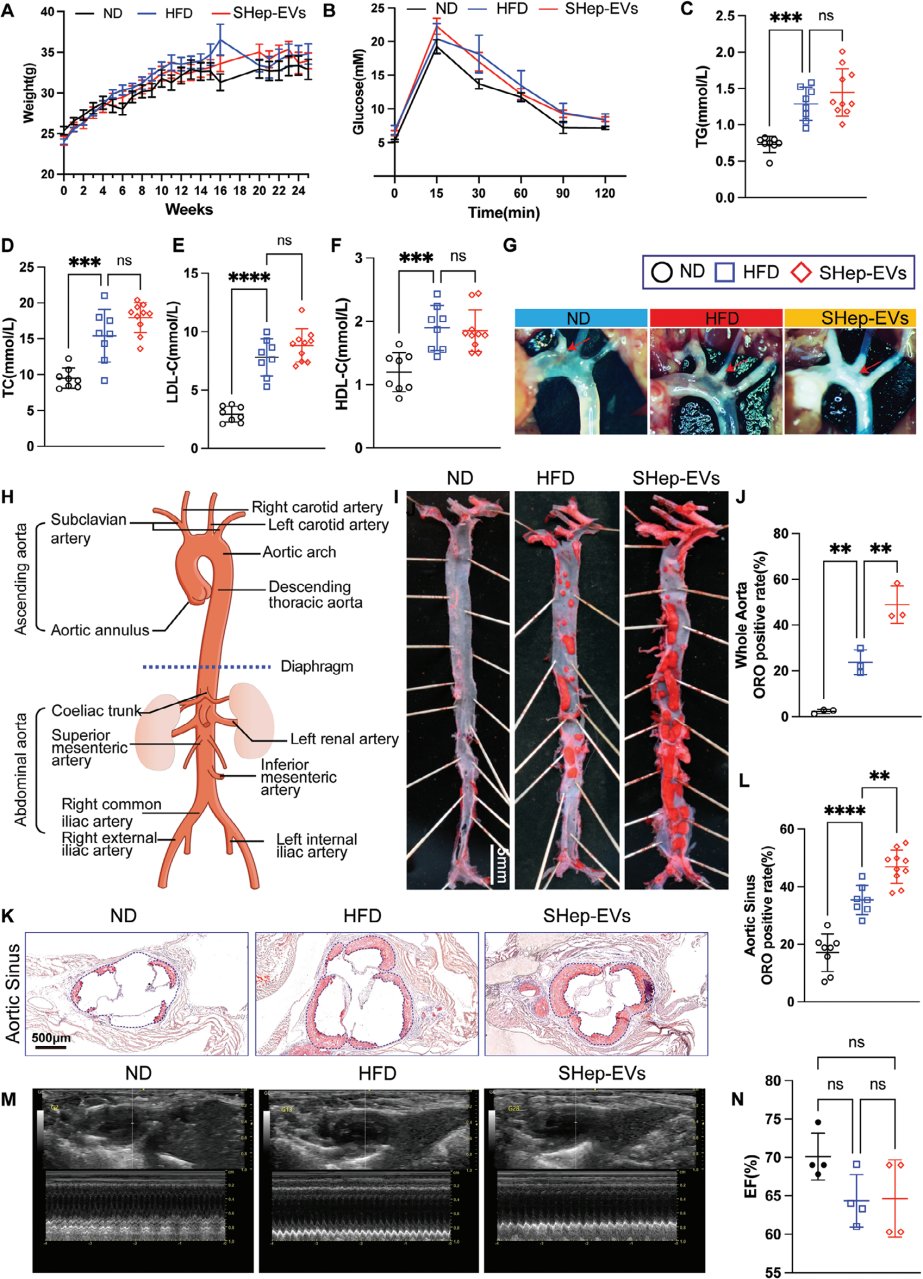
SHep‐EV accelerates atherosclerosis in ApoE^−/−^ mice. A) Weight analysis (*n* = 8 or 10); B) Glucose tolerance test (*n* = 8); C–F) Blood lipid analysis (*n* = 8 or 10); G) Atherosclerotic plaques (white areas) were imaged with a stereomicroscope; H) Schematic diagram of the whole aorta; I,J) ORO staining of the whole aorta and statistical analysis of the percentage of lesion area to the whole aorta area (*n* = 3), scale bar = 5 mm; K,L) Aortic sinus ORO staining and statistical analysis of the percentage of atherosclerotic plaque area (demarcated with a dotted line) to arterial lumen area (*n* = 7–10), scale bar = 500 µm; M,N) Cardiac ultrasound and corresponding statistical analysis (*n* = 4). Data are means ± SD, and *p* values were determined by one‐way ANOVAs followed by Tukey's test; ns: no significant difference, *p < 0.05, **p < 0.01, ***p < 0.001, and ****p < 0.0001. ND: regular diet; HFD: high‐fat diet (D12108C); SHep‐EVs: HFD with 75 µg of SHep‐EVs injected weekly via the tail vein for a total of 24 weeks; EF: left ventricular ejection fraction.

Calcification is a feature of atherosclerosis associated with adverse cardiovascular events.^[^ ^30^ ^]^ To clarify whether SHep‐EVs affect atherosclerotic vascular calcification (intimal calcification), a model of vascular intimal calcification was constructed by feeding ApoE^−/−^ mice a high‐fat diet (R&D12108C) for 24 weeks.^[^ ^31^ ^]^ The experiment was divided into normal diet (ND), high‐fat diet (HFD), and HFD plus SHep‐EV (75 µg of SHep‐EV were injected weekly via the tail vein for a total of 24 weeks) groups. The results showed that the SHep‐EV group had no significant effect on body weight, glucose tolerance, or blood lipid levels (**Figure** **3**A–F). Stereomicroscopy (Figure 3G) and ORO staining of the whole aorta (Figure 3H–J) showed that the positive ORO staining area in the SHep‐EV group was markedly higher than that in the HFD group. We further evaluated atherosclerotic plaques in the aortic arch (Figure S7A,B, Supporting Information), thoracic aorta (Figure S7C,D, Supporting Information), abdominal aorta (Figure S7E,F, Supporting Information), and aortic sinus (Figure 3K,L) by ORO staining. These results suggested that SHep‐EVs significantly increase atherosclerotic plaque formation, particularly in the aortic arch, aortic sinus, and abdominal aorta. In addition, atherosclerotic plaques in the SHep‐EV group were more unstable, exhibiting lower collagen content and larger necrotic cores (Figure S8A–C, Supporting Information). We further assessed the influence of SHep‐EVs on cardiac function through cardiac ultrasound analyses, which revealed that although SHep‐EVs increased atherosclerotic lesions, there was no significant difference in cardiac function (Figure 3M,N).

**Figure 4:**
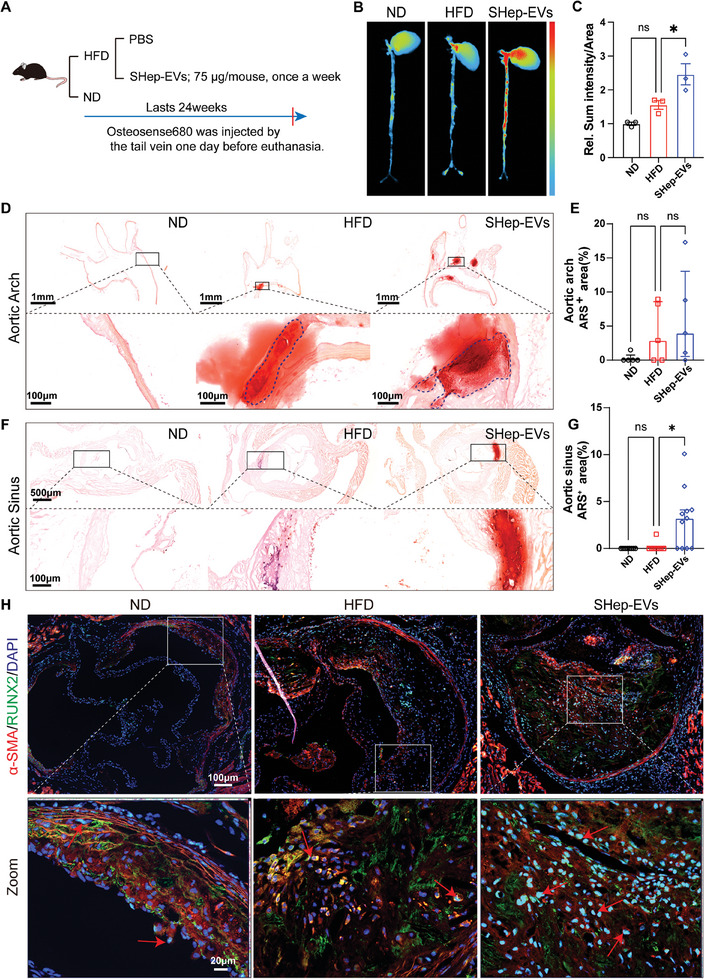
SHep‐EVs accelerate intimal calcification in ApoE^−/−^ mice. A) Schematic diagram of the experimental process: Osteosense 680 EX (100 µL per mouse) was injected into the tail vein 24 h before euthanasia, and the intact heart and aorta of mice were isolated, then fluorescent images were acquired using an in vivo imaging system; B,C) OsteoSense 680 analysis of calcification levels (*n* = 3); D,E) ARS staining and statistical analysis of the aortic arch (*n* = 5), scale bar = 1 mm (top) and 100 µm (bottom); F,G) ARS staining and statistical analysis of the aortic sinus (*n* = 7–10), scale bar = 500 µm (top) and 100 µm (bottom); H) Immunofluorescence analysis of RUNX2 and α‐SMA in the aortic sinus, scale bar = 100 µm (top) and 20 µm (bottom). Data are means ± SD (C), median (interquartile range) (E,G), and *p* values were determined by one‐way ANOVAs followed by Tukey's test; ns: no significant difference, *p < 0.05.

Next, the influence of SHep‐EV on intimal calcification is analyzed by using a near‐infrared bisphosphonate‐based calcium tracer (OsteoSense 680, Perkin Elmer) to analyze arterial calcification severity.^[^ ^31b^ ^]^ OsteoSense 680 fluorescence reflectance imaging showed strong nanoparticle‐derived signals in the arteries of the SHep‐EV‐treated group (**Figure** **4**A–C). ARS staining results indicated that there was no apparent variation in the rate of ARS positivity in the aortic arch (Figure 4D,E). However, a notable increase was observed in the aortic sinus in animals from the SHep‐EV‐treated group. In the HFD group, only a few small punctate calcifications were observed, while in the SHep‐EV group, more macro calcifications were present (Figure 4F,G). Additionally, aortic sinus immunofluorescence revealed that SHep‐EV significantly reduced the expression of the smooth muscle cell contraction phenotypic marker α‐SMA, but increased the expression of the osteogenic marker RUNX2 (Figure 4H), indicating that SHep‐EVs could promote atherosclerosis and intimal calcification.

### 2.4 Inhibition of Hepatic EV Secretion Attenuates MAFLD‐Associated Atherosclerosis and Calcification

**Figure 5:**
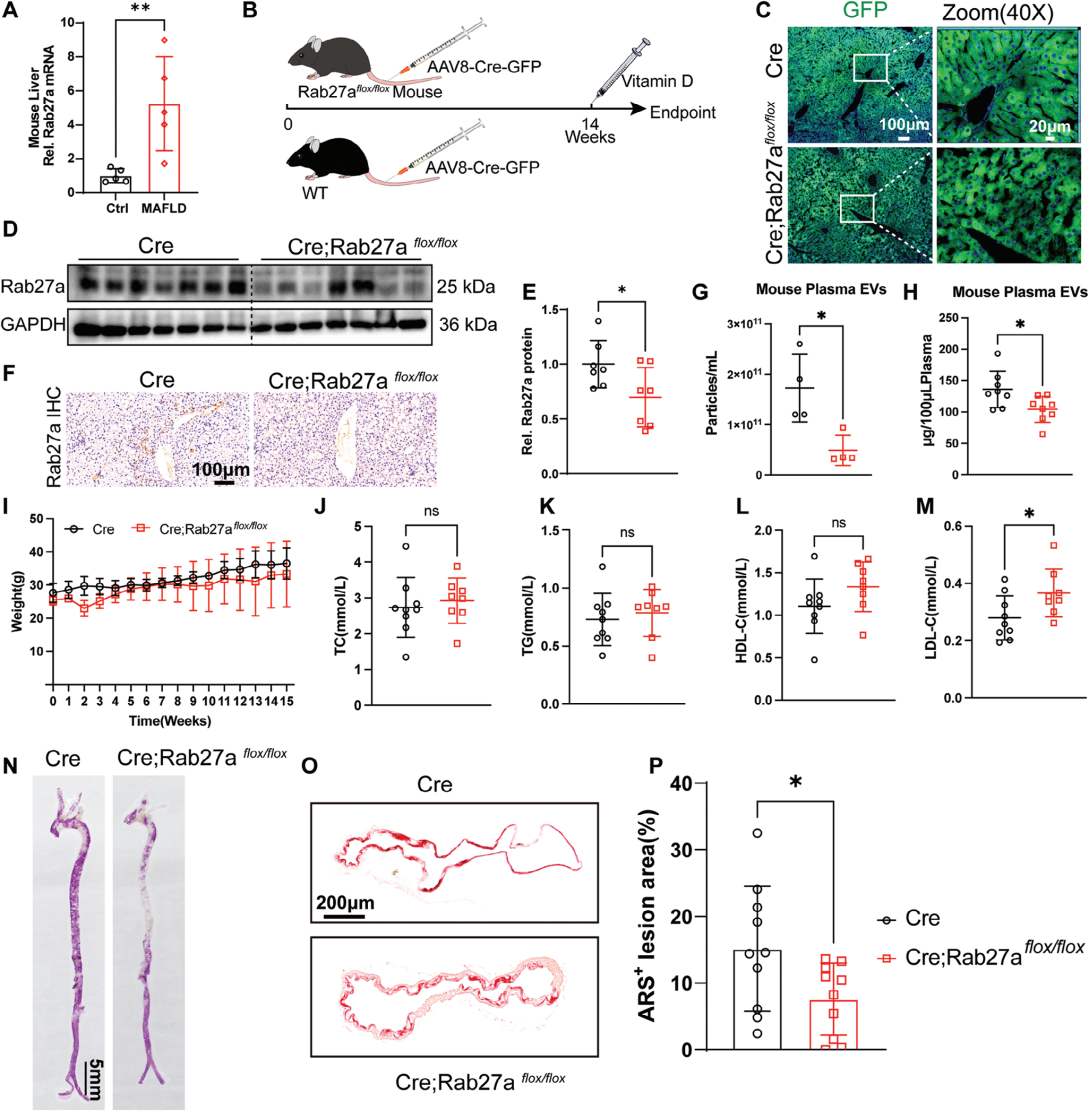
Inhibition of SHep‐EV secretion attenuates MAFLD‐associated vascular calcification. A) Detection of the Rab27a mRNA levels in the murine liver (*n* = 5); B) Schematic diagram of the experimental process; C) Green fluorescent protein expression in liver tissue was detected by immunofluorescence, scale bar = 100 µm (left) and 20 µm (right); D,E) Detection and quantitative analysis of Rab27a protein expression in liver tissue samples; F) Rab27a protein expression in mouse liver tissue samples was detected immunohistochemistry, scale bar = 100 µm; G) Nanoparticle tracking analysis (NTA) analysis of murine plasma EV levels (*n* = 4); H) Analysis of murine plasma EV content by bicinchoninic acid (BCA) method (*n* = 8); I) Mouse body weight (*n* = 8 or 9); J–M) Blood lipid levels (*n* = 8 or 9); N) Whole aortic ARS staining; O,P) ARS staining and corresponding statistical analysis of aortic sections (*n* = 8), scale bar = 200 µm. Data are means ± SD, and *p* values were determined by unpaired two‐tailed Student's t‐tests; ns: no significant difference, *p < 0.05, **p < 0.01.

To further validate that EVs released from steatotic hepatocytes play a significant role in MAFLD‐associated vascular calcification, we created mice in which liver‐specific EV release was blocked through the liver‐specific knockout of Rab27a. Rab27a is a key protein that mediates the secretion of EVs.^[^ ^32^ ^]^ Consistent with previous reports,^[^ ^33^ ^]^ Rab27a expression in the liver tissue was increased in MAFLD mice (**Figure** **5**A). Rab27a was deleted in the liver through the tail vein injection of a liver‐specific Cre expression adeno‐associated virus with an ApoE/hAATp promoter into Rab27a*^flox/flox^* mice (Figure S9, Supporting Information). In parallel, AAV‐Cre‐GFP was injected into wild‐type (WT) mice to generate a control group (Figure 5B).

The extent of virus coverage was confirmed by GFP expression (Figure 5C). Western blotting of total liver tissue lysates taken 14 weeks later revealed obvious reduction in Rab27a protein levels in the liver of AAV‐Cre‐GFP‐injected Rab27a*^flox/flox^* mice (Cre; Rab27^flox/flox^) compared with WT mice (Figure 5D,E). Immunohistochemical staining of liver tissue also showed that the rate of Rab27a positivity in the Cre;Rab27*^flox/flox^* group was significantly reduced (Figure 5F). Moreover, liver‐specific knockdown of Rab27a significantly reduced the contents of EVs in murine plasma (Figure 5G,H). Liver‐specific knockdown of Rab27a had no significant effect on mouse body weight (Figure 5I), but blood lipid levels were slightly elevated (Figure 5J–M). Immunohistochemical staining results demonstrated that inhibition of SHep‐EV secretion reduces the expression of the osteogenic marker RUNX2 in the aorta of MAFLD mice (Figure S10, Supporting Information). In addition, the whole aorta and aortic section ARS staining (Figure 5N–P) demonstrated that the liver‐specific blockade of EVs release attenuated MAFLD‐associated vascular calcification.

**Figure 6:**
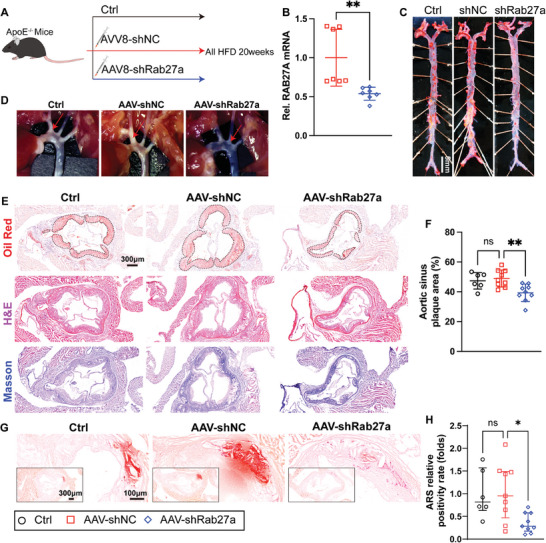
Inhibition of SHep‐EV secretion attenuates MAFLD‐associated atherosclerosis and calcification. A) Schematic diagram of the experimental process; B) Detection of Rab27a mRNA levels in murine liver tissue (*n* = 7); C) ORO staining of the whole aorta, scale bar = 5 mm; D) Aortic arch atherosclerotic plaques were imaged under a stereomicroscope (white areas); E) Aortic sinus ORO, H&E, and Masson staining, scale bar = 300 µm; F) ORO staining statistics of the aortic sinus (*n* = 6 or 9); G,H) ARS staining of the aortic sinus and corresponding statistical analysis (*n* = 6 or 8), scale bar = 300 µm and 100 µm. Data are presented as the mean ± SD (6 B, F) or median (interquartile range) (H), and *p* values were determined by unpaired two‐tailed Student's t‐tests (B) or one‐way ANOVAs followed by Tukey's test (F, H); ns: no significant difference, *p < 0.05, and **p < 0.01.

To determine whether EVs secreted by hepatocytes also play an important role in atherosclerosis and associated calcification. We established a liver‐specific knockdown Rab27a model in ApoE^−/−^ mice through the injection of a liver‐specific shRab27a adeno‐associated virus (**Figure** **6**A), and liver tissue mRNA analysis showed that this virus significantly down‐regulated Rab27a expression by ≈50% (Figure 6B). Liver‐specific knockdown of Rab27a in ApoE^−/–^ mice slightly reduced murine body weight (Figure S11A, Supporting Information) and partially lowered blood lipid levels, especially those of LDL‐c (Figure S11B–E, Supporting Information), but had no significant effect on liver function (Figure S11F,G, Supporting Information). ORO staining of the whole aorta (Figure 6C), aortic arch microscopy (Figure 6D), and aortic sinus ORO staining (Figure 6E,F) showed that inhibition of Rab27a expression in hepatocytes significantly attenuated atherosclerosis. ARS staining showed that inhibition of Rab27a expression in the liver could also reduce atherosclerotic plaque calcification (Figure 6G,H). These results suggest that SHep‐EVs at least partially mediate MAFLD‐associated atherosclerosis and calcification.

### 2.5 SHep‐EVs Promote the Osteogenic Differentiation of VSMCs In Vitro

**Figure 7:**
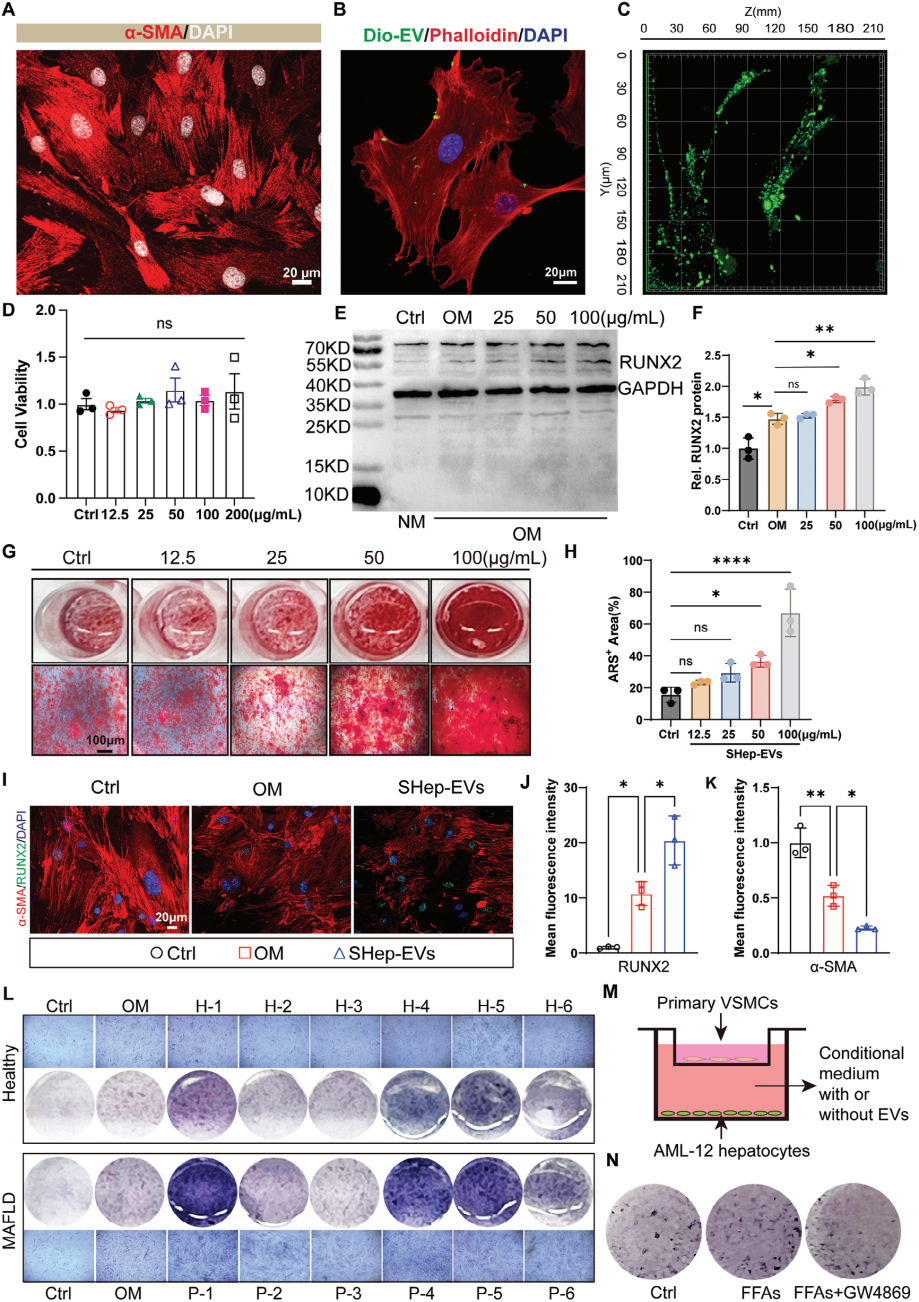
SHep‐EVs promote the osteogenic differentiation of VSMCs in vitro. A) Immunofluorescence analysis of mouse primary aortic smooth muscle cells (α‐SMA in red, nuclei in white), scale bar=20 µm; B) Analysis of SHep‐EV uptake by vascular smooth muscle cells(VSMCs) (green indicates DiO‐labeled SHep‐EVs at a concentration of 50 µg mL^−1^ incubated for 4 h; red indicates the phalloidin‐labeled cytoskeleton), scale bar = 20 µm; C) Fluorescence reconstruction of DiO‐labeled SHep‐EVs after co‐incubation with VSMCs for 12 h; D) The effect of different concentrations of SHep‐EVs on the viability of VSMCs (*n* = 3); E,F) Western blotting detection and quantitative analysis of the expression of the osteogenic marker RUNX2 in VSMCs treated with different concentrations of SHep‐EVs for 7 days; G,H) ARS staining and statistical analysis of VSMCs treated with different concentrations of SHep‐EVs for 21 days (*n* = 3); I–K) Immunofluorescent staining and statistical analysis of RUNX2 (green) and α‐SMA (red) following the treatment of VSMCs with 100 µg mL^−1^ of SHep‐EVs for 7 days (*n* = 3), scale bar=20 µm; L) ALP staining of VSMCs treated with plasma EVs (100 µg mL^−1^) from 6 independent healthy or MAFLD patients for 7 days; M) Schematic diagram of a co‐culture assay in which normal hepatocytes or steatotic hepatocytes with or without exosomes were co‐cultured with VSMCs in 12‐well transwell inserts; N) ALP staining of VSMCs was performed in the upper chamber after 3 days of co‐culture. Data are means ± SD, and *p* values were determined by one‐way ANOVAs followed by Tukey's test; ns: no significant difference, *p < 0.05, **p < 0.01, and ****p < 0.0001.

Physiologically, VSMCs are located in the middle layer of the arteries, present a contractile phenotype, and express high levels of α‐SMA, SMMHC, and SM‐22α, which are responsible for the maintenance of vascular morphology and normal diastolic and contractile functions. Pathologically, VSMCs can transform to a proliferative phenotype, migrate to the intima, and secrete large quantities of extracellular matrix proteins.^[^ ^34^ ^]^ Studies have shown that the osteogenic differentiation of VSMCs is a key factor in the progression of vascular calcification.^[^ ^35^ ^]^ To clarify whether SHep‐EVs promote calcification by inducing the osteogenic differentiation of VSMCs, we first isolated primary mouse aortic smooth muscle cells. Immunofluorescence analysis showed that the isolated primary mouse aortic smooth muscle cells highly expressed the smooth muscle cell marker α‐SMA, indicating the successful isolation of high‐purity mouse aortic smooth muscle cells (**Figure** **7**A). After incubating DiO‐labeled SHep‐EVs with VSMCs for 4 h, labeled EVs could be observed in the cytoplasm of VSMCs (Figure 7B). After 12 h, more abundant DiO‐labeled EVs were observed in VSMCs, indicating that VSMCs can take up SHep‐EVs (Figure 7C).

SHep‐EVs delivered at concentrations of 0–200 µg mL^−1^ had no significant effect on VSMC viability (Figure 7D), but did promote the osteogenic differentiation of VSMCs in a concentration‐dependent manner. Based on the results of RUNX2 and BMP2 protein expression analyses (Figure 7E,F) (Figure S12A,B, Supporting Information) and ARS staining (Figure 7G,H), we finally chose 100 µg mL^−1^ as the SHep‐EV treatment concentration in this study. Immunofluorescence results showed that SHep‐EVs significantly inhibited the expression of α‐SMA but increased the expression of RUNX2 (Figure 7I–K). Furthermore, we compared the effects of plasma EVs from healthy individuals and baseline‐matched MAFLD patients (Table S4, Supporting Information) on the osteogenic differentiation of VSMCs. The results of ALP staining showed that plasma EVs from MAFLD patients had a stronger ability to promote the osteogenic differentiation of VSMCs (Figure 7L). To investigate whether EVs are involved in cell‐cell communication between hepatocytes and VSMCs, a transwell co‐culture assay was conducted in which the two cell types were separated by a membrane with a 0.4 µm pore size, thus preventing direct contact but allowing EVs to pass freely through the membrane (Figure 7M). This experiment revealed that VSMCs co‐cultured with hepatocytes treated with FFAs had a notably higher positive rate of ALP staining. Conversely, ALP staining positivity was reduced when VSMCs were co‐cultured with steatotic hepatocytes treated with the exosome release inhibitor GW4869 (Figure 7N), suggesting a critical role for SHep‐EVs in this process.

Moreover, we compared the effects of Hep‐EVs and SHep‐EVs on VSMCs calcification by assessing the protein (Figure S12C,D, Supporting Information) and mRNA levels (Figure S12E,F, Supporting Information) of VSMC‐related markers (α‐SMA, SM‐22α) and osteogenic differentiation markers (RUNX2, BMP2). We also performed ALP staining (Figure S12G, Supporting Information) and activity assays (Figure S12H, Supporting Information). While Hep‐EVs had no significant effect on VSMC osteogenic differentiation, SHep‐EVs induced VSMC calcification. Collectively, the above data suggest that steatotic hepatocytes potently promote the osteogenic differentiation of VSMCs through the release of EVs.

### 2.6 SHep‐EVs Induce M1 Macrophage Polarization and Promote Foam Cell Formation

We additionally studied the molecular mechanisms by which SHep‐EVs promote atherosclerosis. The etiology of atherosclerosis is complex, and multiple cell types are involved in forming atherosclerotic plaques, including endothelial cells, VSMCs, dendritic cells, and macrophages.^[^ ^36^ ^]^ Macrophages and cholesterol‐rich foam cells in the arterial intima are hallmarks of atherosclerotic progression.^[^ ^37^ ^]^ In addition, macrophages exhibit strong plasticity and can be polarized into two different functional subsets in the context of the atherosclerotic inflammatory response, including pro‐inflammatory M1 and anti‐inflammatory M2 cells.^[^ ^38^ ^]^ M1 macrophages are known to secrete matrix metalloproteinases (MMPs), such as MMP2 and MMP9, which can lead to degradation of the extracellular matrix in plaques, causing plaque instability and rupture, thereby increasing the risk of acute cardiovascular events. EVs derived from steatotic hepatocytes have been observed to promote atherosclerosis by inducing endothelial inflammation.^[^ ^19^ ^]^ Analyses of murine plasma inflammatory factors revealed that levels of the inflammatory factor IL‐1β were significantly increased in the SHep‐EVs group, but remained unclear as to whether SHep‐EVs can promote macrophage proliferation and phenotypic transformation. In this study, we thus primarily focused on the roles of macrophages and foam cells.

**Figure 8:**
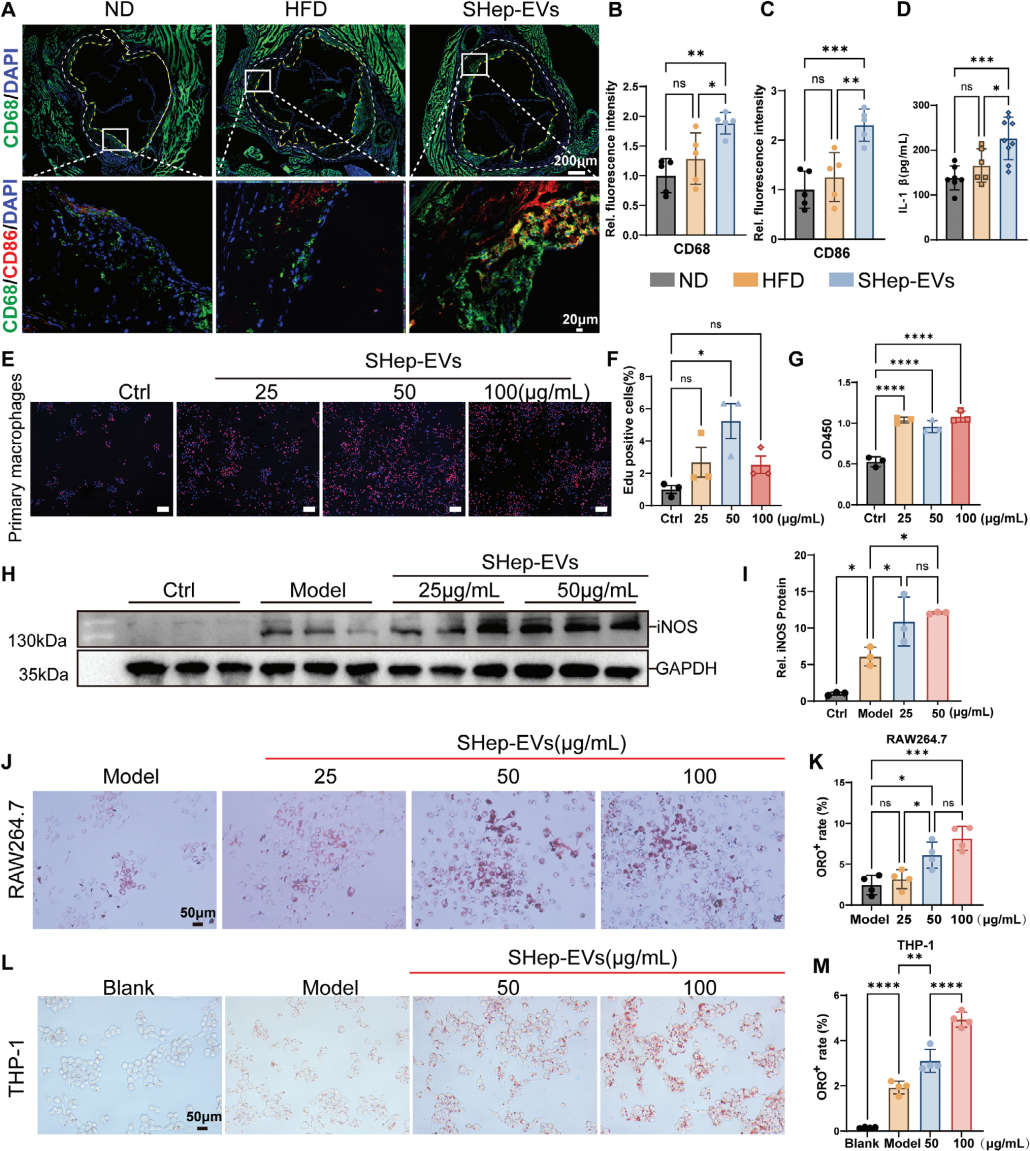
SHep‐EVs induce M1 macrophage polarization and promote foam cell formation. A–C) Immunofluorescence analysis of the expression of the macrophage markers CD68 and CD86 in atherosclerotic plaques (*n* = 5), with CD68 in green, CD86 in red, and DAPI‐labeled nuclei in blue, scale bar = 200 µm (top) and 20 µm (bottom), NOTE: CD86 fluorescence signal was only acquired at 40×; D) Plasma IL‐1β levels in mice were measured by ELISA (*n* = 6**–**9); E,F) Macrophage proliferation ability was analyzed in an EdU assay, with the red fluorescent signal stemming from EdU incorporation into the DNA during DNA synthesis enabling the assessment of cellular proliferation by counting EdU‐positive cells (*n* = 3), scale bar = 100 µm; G) Macrophage viability was detected by CCK‐8 assay (*n* = 3); H,I) The expression of the M1 macrophage marker iNOS was analyzed by western blotting (*n* = 3), J,K) RAW264.7 macrophage ORO staining; L,M) THP‐1 macrophage ORO staining. Data are means ± SD, and *p* values were determined by one‐way ANOVAs followed by Tukey's test; ns: no significant difference, *p < 0.05, **p < 0.01, ***p < 0.001, and ****p < 0.0001. Ctrl: blank control, Model group: IFN‐γ (10 ng mL^−1^) + LPS (100 ng mL^−1^) to simulate macrophage inflammation in the atherosclerotic state, SHep‐EVs groups refer to the SHep‐EVs administered together with model group treatment for 24 h.

First, we analyzed the expression of macrophage‐associated markers in atherosclerotic plaques and found that the total number of macrophages (CD68+ cells) in atherosclerotic plaques was increased in the SHep‐EV group, and the expression of the M1‐type macrophage marker, CD86, was increased (**Figure** **8**A–C). In addition, SHep‐EVs significantly increased plasma IL‐1β levels in ApoE^−/−^mice (Figure 8D). Subsequently, we treated primary mouse macrophages with different concentrations of SHep‐EVs. Through EdU analysis experiments and cell viability analyses, we found that SHep‐EVs were able to significantly promote macrophage proliferation (Figure 8E–G). To further clarify the role of SHep‐EVs in macrophages, we co‐treated macrophages with IFN‐γ (10 ng mL^−1^) + LPS (100 ng mL^−1^) for 24 h to simulate the inflammatory state of macrophages in the context of atherosclerosis.^[^ ^39^ ^]^ (Model group), and simultaneously treated these cells with different concentrations of SHep‐EVs (SHep‐EV group). SHep‐EVs promoted iNOS upregulation in macrophages in a concentration‐dependent manner (Figure 8H,I), indicating that SHep‐EVs can promote the M1 polarization of macrophages.

Finally, we analyzed the effect of SHep‐EVs on macrophage lipid loading. Consistent with a recent report,^[^ ^29^ ^]^ SHep‐EVs increased RAW264.7 and THP‐1 macrophage lipid loading in a concentration‐dependent manner (Figure 8J–M). In summary, these results indicate that SHep‐EVs increase systemic and local vascular inflammation in ApoE^−/−^ mice. In light of these data, we speculate that the role of SHep‐EVs in promoting atherosclerosis may be related to their systemic pro‐inflammatory effects, promoting macrophage proliferation and M1 polarization while increasing macrophage lipid loading.

### 2.7 Changes in the Expression of EVs Proteins Derived from MAFLD Mouse Primary Hepatocytes

**Figure 9:**
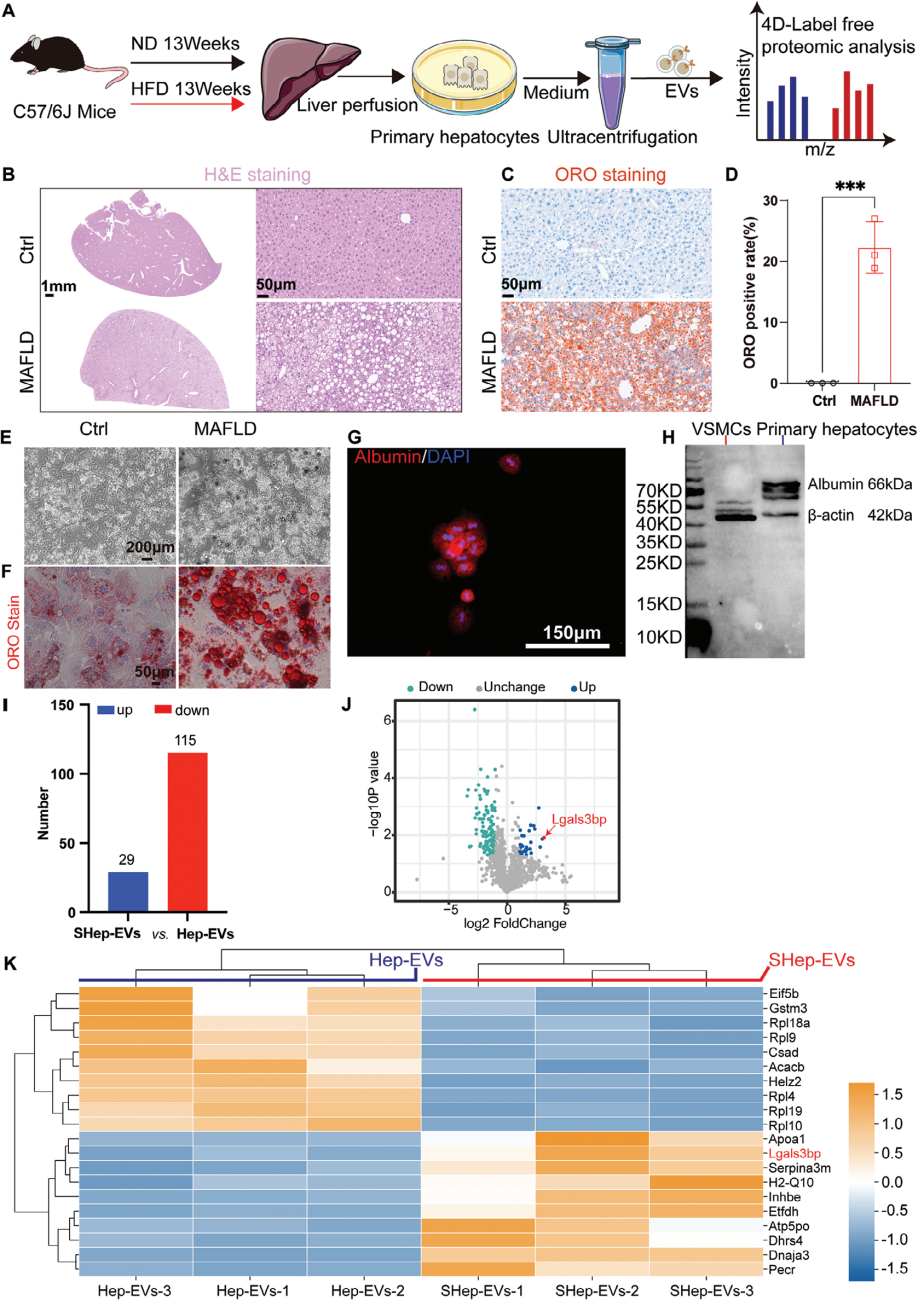
Primary hepatocytes‐derived EVs proteomic analysis. A) Schematic diagram of the experimental process; B) H&E staining of liver tissue sections, scale bar = 1 mm(left) or 50 µm(right); C,D) ORO staining of liver tissue sections and the statistical analysis (*n* = 3), scale bar = 50 µm. Data are presented as mean ± SD, *p* values were determined by unpaired two‐tailed Student's t‐test, “***” represents *p *< 0.001; E) Morphological comparison of primary hepatocytes under a light microscope, scale bar = 200 µm; F) ORO staining of primary hepatocytes, scale bar = 50 µm; G) Albumin expression be detected by immunofluorescence; H) The expression of albumin in VSMCs and primary hepatocytes be detected by western blot; I) The total number of differential proteins be identified; J) Volcano map of differential proteins; K) Top 10 differential protein heat map. Protein expression differences >2‐fold and the *p‐value* <0.05 were considered differentially expressed proteins.

To identify the components responsible for the pro‐atherosclerotic and vascular calcification effects of SHep‐EVs, we first established a murine MAFLD model by feeding mice with HFD (**Figure** **9**A; Figure S13, Supporting Information). After 13 weeks, mice were visibly obese and had gained weight (Figure S13A,B, Supporting Information), accompanied by abnormal glucose tolerance (Figure S13C, Supporting Information) and increased plasma AST and blood lipid levels (Figure S13D–H, Supporting Information). H&E and ORO staining (Figure 9B–D) revealed the presence of numerous vacuoles and lipid deposits in the liver tissue sections of MAFLD mice. These results indicated that we successfully constructed a mouse model of MAFLD. We then extracted the primary hepatocytes and there derived EVs for proteomic analysis (Figure S14, Supporting Information). The primary hepatocytes were able to adhere to the culture plate wall 4 h after separation, and these hepatocytes were binucleate and hexagonal (Figure S13C, Supporting Information). Compared with the primary hepatocytes from mice fed a normal diet, an increase in the presence of lipid droplets was observed in MAFLD mice, and ORO staining made this difference more obvious (Figure 9E,F). Albumin is highly expressed in healthy functional hepatocytes.^[^ ^40^ ^]^ Western blot revealed that VSMCs did not express albumin, whereas primary hepatocytes expressed it in abundance (Figure 9H). This was further confirmed by immunofluorescence (Figure 9G). These results indicate that we successfully constructed a murine MAFLD model and were able to obtain high‐purity hepatocytes from these animals.

Based on the above results, we separated EVs from primary hepatocytes by ultracentrifugation for 4D label‐free proteomics analyses. In total, 144 differential proteins were identified, including 29 up‐regulated proteins and 115 down‐regulated proteins (Figure 9I,J). These proteins were primarily related to lipid metabolism and the extracellular matrix. Among these proteins, Lgals3bp was the most highly upregulated (Figure 9J,K). Through a literature review, we found that Lgals3bp is highly enriched in EVs.^[^ ^41^ ^]^ More importantly, it has been reported that Lgals3bp can promote the osteogenic differentiation of human periodontal ligament stem cells (hPDLSCs),^[^ ^42^ ^]^ suggesting that it may serve as a mediator of the ability of SHep‐EVs to promote vascular calcification.

**Figure 10:**
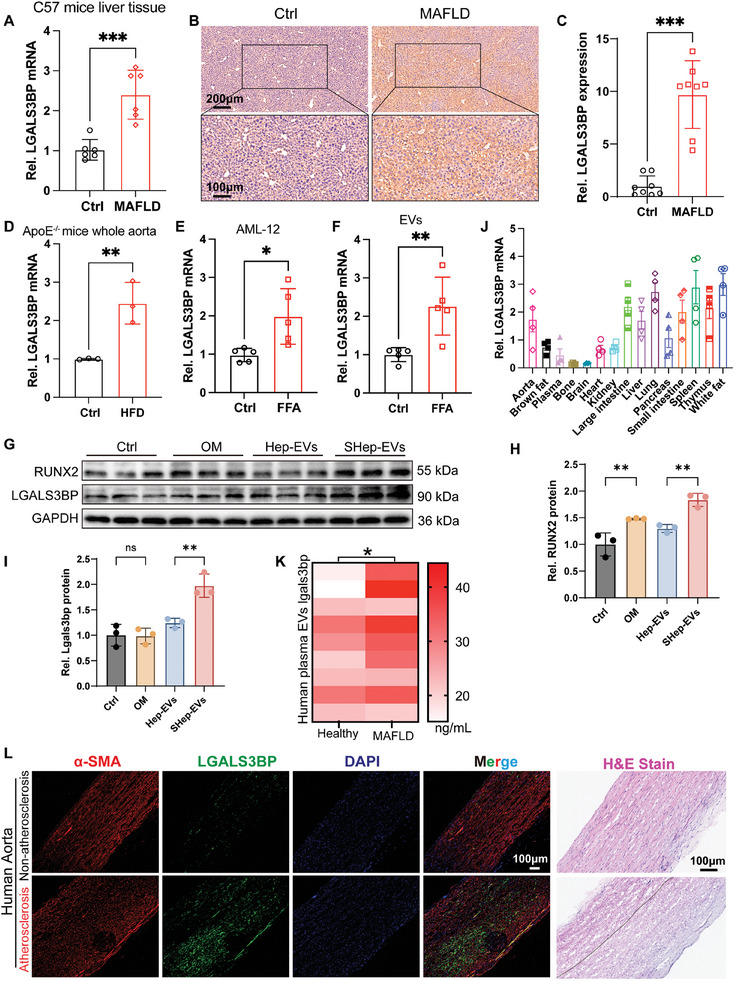
Analysis of Lgals3bp expression. A) Lgals3bp mRNA levels in mouse liver tissue(*n* = 6); B,C) Immunohistochemical analysis of Lgals3bp protein expression levels in mouse liver tissue (*n* = 8), scale bar = 200 µm (top) or 100 µm (bottom); D) Lgals3bp mRNA expression levels in the aorta of ApoE^−/‐^ mice(*n* = 3); E) Lgals3bp mRNA expression levels in AML‐12 hepatocytes (*n* = 5); F) Lgals3bp mRNA expression levels in AML‐12 hepatocyte‐derived EVs (*n* = 5); G–I) Protein expression level of Lgals3bp in VSMCs (*n* = 3); J) Tissue expression profile of Lgals3bp in 8‐week‐old male C57/6J mice (*n* = 4); K) Protein expression of Lgals3bp in human plasma‐derived EVs (*n* = 9); L) Immunofluorescence and H&E staining of Lgals3bp and α‐SMA in human aorta, scale bar = 100 µm. Data are presented as mean±SD, *p* values were determined by unpaired two‐tailed Student's t‐test, “ns” represents no significant difference, “*” represents *p *< 0.05, “**” represents *p *< 0.01, and “***” represents *p *< 0.001.

To verify this hypothesis, we first measured the expression of Lgals3bp in the liver of mice. Both qPCR and immunohistochemistry results showed that HFD feeding significantly upregulated hepatic Lgals3bp expression (**Figure** **10**A–C). In addition, Lgals3bp levels were elevated in the aorta of HFD‐fed ApoE^−/−^ mice (Figure 10D), as well as in hepatocytes treated with FFAs and their derived EVs (Figure 10E,F), and in SHep‐EV‐treated VSMCs (Figure 10G–I). Next, we detected the expression profile of Lgals3bp in mice, and found that Lgals3bp was expressed in multiple tissues, with high expression levels in the liver (Figure 10J).

Finally, we detected the expression of Lgals3bp in the human aorta, plasma‐derived EVs, and found that compared with healthy individuals, the expression of Lgals3bp was up‐regulated in EVs derived from the plasma of patients with MAFLD (Figure 10K), and that the protein levels of Lgals3bp were elevated in human atherosclerotic lesions (Figure 10L), consistent with previous reports.^[^ ^43^ ^]^ Collectively, these data suggest that Lgals3bp‐rich EVs potentially serve to promote atherosclerosis and calcification.

### 2.8 Lgals3bp Knockdown in Hepatocytes Inhibits SHep‐EV‐Induced VSMC Osteogenic Differentiation

**Figure 11:**
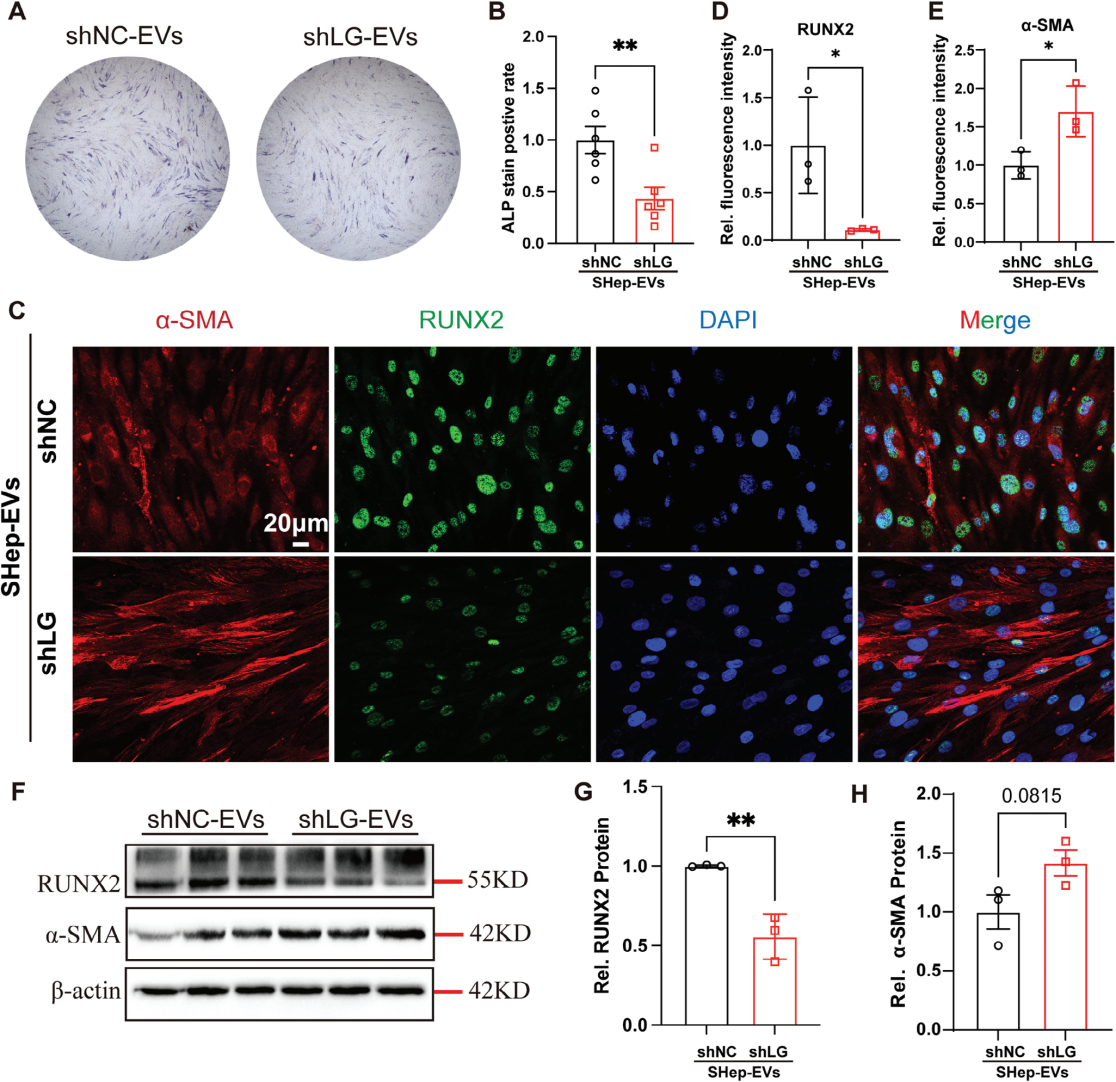
Hepatocyte‐specific deficiency Lgals3bp inhibits vascular calcification in MAFLD mice. A,B) ALP staining and statistical analyses were performed 7 days after treatment of hVSMCs with EVs derived from AML‐12 hepatocyte culture medium using transfected negative control or knockdown of Lgals3bp shRNA (*n* = 6); C–E) Immunofluorescence analysis of the expression of RUNX2 and α‐SMA in hVSMCs were performed 7 days after treatment of hVSMCs with EVs derived from AML‐12 hepatocyte culture medium using transfected negative control or knockdown of Lgals3bp shRNA (*n* = 3), scale bar = 20 µm; F–H) The protein expressions of RUNX2 and α‐SMA were analyzed by western blotting 7 days after treatment of hVSMCs with EVs derived from AML‐12 hepatocyte culture medium using transfected negative control or knockdown of Lgals3bp shRNA (*n* = 3). Data are presented as mean ± SD, *p* values were determined by unpaired two‐tailed Student's t‐test, “*” represents *p *< 0.05, and “**” represents *p *< 0.01.

To further clarify the effect of Lgals3bp in SHep‐EVs on the osteogenic differentiation of VSMCs, we constructed a Lgals3bp‐specific short hairpin RNA (shLgals3bp, shLG). Transfected hepatocytes exhibited a strong eGFP signal (Figure S15A, Supporting Information). Both qPCR and Western blotting results showed that the knockdown efficiencies of Lgals3bp shRNA‐2 and ‐3 were up to ≈80% and 50% at mRNA and protein levels, respectively (Figure S15B,C, Supporting Information), with shRNA‐2 performing more reproducibly. Therefore, Lgals3bp shRNA‐2 was used in subsequent experiments. Lgals3bp knockdown hepatocyte‐derived EVs significantly reduced the rate of ALP positivity in osteogenically induced VSMCs compared with the shRNA negative control transfection group (**Figure** **11**A,B), and immunofluorescence staining revealed that the knockdown of Lgals3bp significantly inhibited RUNX2 expression but increased the expression of α‐SMA (Figure 11C–E). Western blotting results were consistent with these immunofluorescence results (Figure 11F–H), suggesting that hepatocytes Lgals3bp deficiency inhibits the SHep‐EV‐induced osteogenic differentiation of VSMCs and may be a therapeutic target for MAFLD‐related vascular calcification.

### 2.9 Hepatocyte‐Specific Lgals3bp Deficiency Inhibits MAFLD‐Related Vascular Calcification

**Figure 12:**
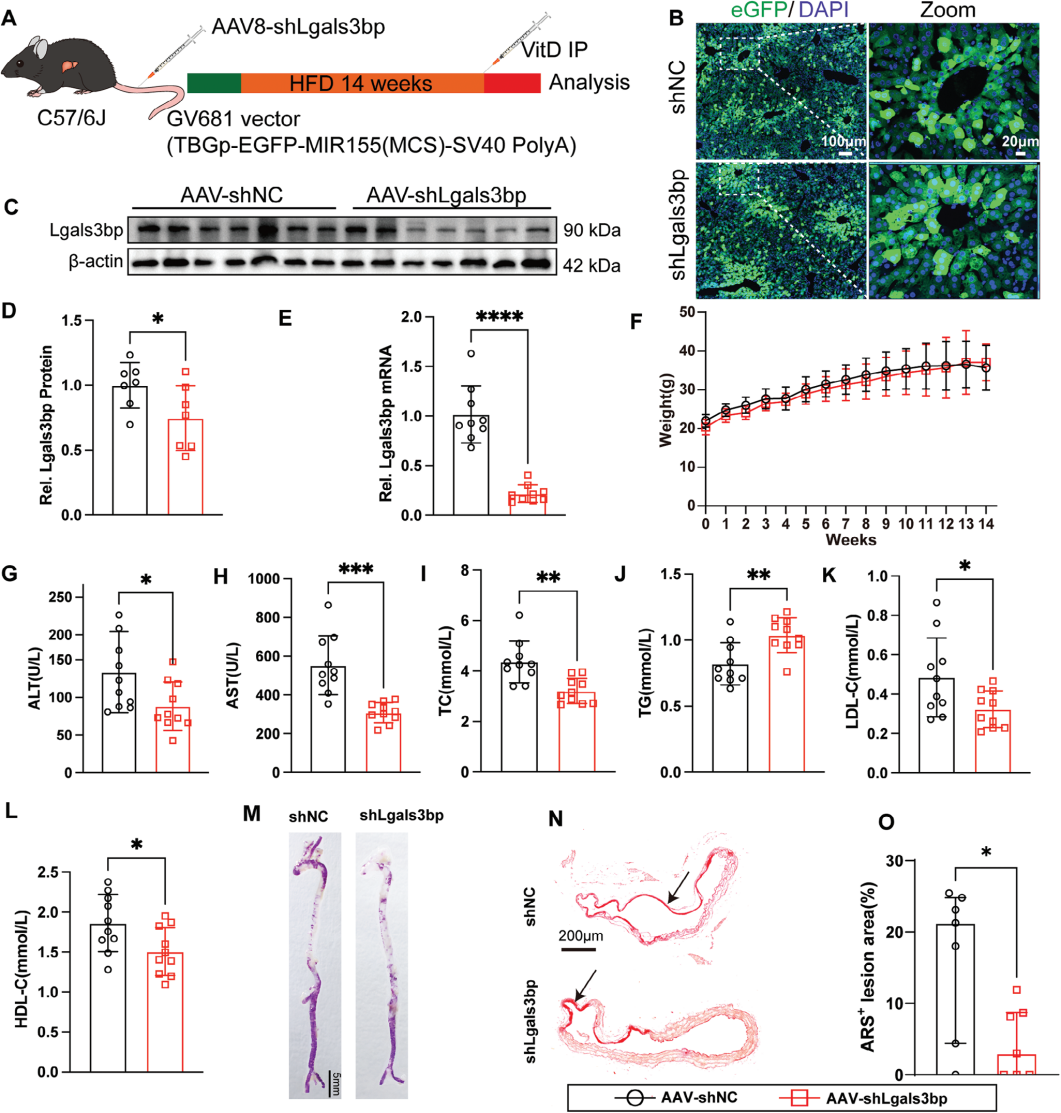
Hepatocyte‐specific deficiency Lgals3bp inhibits vascular calcification in MAFLD mice. A. Schematic diagram of the experimental process; B) GFP expression in liver tissue was detected by immunofluorescence, scale bar = 100 µm(left) and 20 µm(right); C,D) Lgals3bp in liver tissue was also analyzed by western blot (*n* = 7); E) qPCR detected the mRNA levels of Lgals3bp (*n* = 9);F) Mouse body weight(*n* = 12); G,H) liver function index (*n* = 10); I–L) Blood lipid levels (*n* = 10); M) Whole aortic ARS staining, scale bar = 5 mm; N,O) ARS staining and statistics of aortic sections (*n* = 7), scale bar = 200 µm. Data are presented as mean ± SD (12 D‐L) median (interquartile range) (12 O), *p* values were determined by unpaired two‐tailed Student's t‐test(12 D‐L), and nonparametric tests (Mann‐Whitney U test) (12 O), “*” represents *p *< 0.05, “**” represents *p *< 0.01, “***” represents *p *< 0.001 and “****” represents *p *< 0.0001.

To ascertain whether Lgals3bp is the key molecule that mediates liver‐vascular communication to promote MAFLD‐associated atherosclerosis and calcification, we created hepatocyte‐specific Lgals3bp‐knockout mice by injecting them with a liver‐specific knockout adeno‐associated virus (TBGp‐EGFP‐MIR155(MCS)‐SV40 PolyA) via the tail vein (**Figure** **12**A). The extent of viral coverage was confirmed by GFP expression (Figure 12B). The qPCR and western blotting results showed that the knockdown efficiency of Lgals3bp was ≈80% and 40% at the mRNA and protein levels, respectively (Figure 12C–E). Liver‐specific knockdown of Lgals3bp had no significant effect on the body weight of mice (Figure 12F). Interestingly, liver‐specific knockdown of Lgals3bp improved liver function (Figure 12G,H) while also reducing TC and LDL‐c levels (Figure 12I,K). However, it increased plasma TG levels (Figure 12J). ARS staining of the whole aorta and aortic sections (Figure 12M–O) demonstrated that the liver‐specific knockdown of Lgals3bp attenuated MAFLD‐associated vascular calcification. These results suggest that Lgals3bp in SHep‐EVs at least partially mediates MAFLD‐associated vascular calcification.

**Figure 13:**
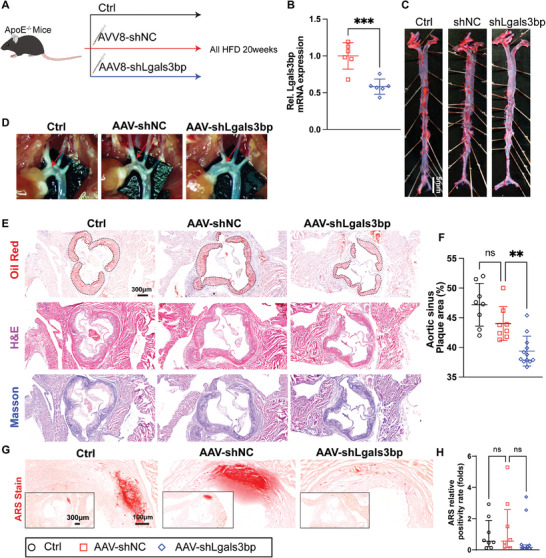
Hepatocyte‐specific deficiency Lgals3bp in ApoE^−/‐^ mice attenuates atherosclerosis and calcification. A) Schematic diagram of the experimental process; B) Detection of the mRNA expression level of Lgals3bp in mouse liver tissue (*n* = 6); C) ORO staining of the whole aorta, scale bar = 5 mm; D) Aortic arch atherosclerotic plaque under stereomicroscope (the white part). E) Aortic sinus ORO, H&E, and Masson staining, scale bar = 300 µm; F) ORO staining statistics of the aortic sinus (*n* = 8 or 12). G,H) ARS staining and statistics of the aortic sinus (*n* = 8 or 10). Data are presented as mean ± SD (13 B, F), or median (interquartile range) (13 H), *p* values were determined by unpaired two‐tailed Student's t‐test(13B) or one‐way ANOVA followed by Tukey's test, “ns” represents no significant difference, “**” represents *p *< 0.01, “***” represents *p *< 0.001.

To ascertain whether Lgals3bp enriched in EVs also plays an important role in atherosclerosis and associated calcification. We constructed a liver‐specific knockdown Lgals3bp model in ApoE^−/−^ mice by injecting a liver‐specific Lgals3bp knockdown adeno‐associated virus (**Figure** **13**A), with qPCR analyses of liver tissue samples confirming that the Lgals3bp knock‐down adeno‐associated virus significantly down‐regulated Lgals3bp expression (Figure 13B). After 12 weeks of HFD feeding, mice in the liver‐specific knockout Lgals3bp group began to demonstrate a trend toward weight gain compared with the NC group (Figure S16A, Supporting Information). Liver‐specific knockdown Lgals3bp has no significant effect on liver function in ApoE^−/‐^ mice (Figure S16B,C, Supporting Information). However, unlike in C57 mice, liver‐specific knockdown of Lgals3bp in ApoE^−/−^ mice increased plasma TC and HDL‐c levels (Figure S16D–G, Supporting Information). ORO staining of the whole aorta (Figure 13C), aortic arch microscopy (Figure 13D), and aortic sinus staining (Figure 13E,F) showed that the inhibition of Lgals3bp expression in hepatocytes significantly attenuated atherosclerosis. ARS staining showed that the inhibition of Lgals3bp expression in the liver could also reduce atherosclerotic plaque calcification (Figure 13G,H). These results suggest that Lgals3bp enriched in SHep‐EVs at least partially mediates MAFLD‐associated atherosclerosis and calcification.

## 3 Discussion

Atherosclerosis and vascular calcification are major causes of death from cardiovascular and cerebrovascular diseases and are predictors of all‐cause mortality. Unfortunately, the pathogenesis of vascular calcification has not been elucidated and few clinical treatments are available, resulting in an unsatisfactory outcome and prognosis. MAFLD affects ≈1/3 of the global population and is a major social public health problem.^[^ ^3^ ^]^ Recent studies have revealed that atherosclerosis and calcification are more prevalent among MAFLD patients, but the underlying mechanisms remain unclear.^[^ ^1^, ^14^ ^]^

Increasing evidence indicates that EVs can mediate the bidirectional transfer of functional molecules (such as proteins, RNA, and lipids) between cells. When EVs are secreted by maternal cells and taken up by target cells through various pathways, their contents are released, thereby affecting the function of the recipient cells. For example, VSMCs in calcification medium release large amounts of EVs with pro‐calcification effects.^[^ ^44^ ^]^ Macrophage‐derived EVs in patients with CKD have been shown to promote the formation of micro‐calcifications in atherosclerotic plaques.^[^ ^31a^ ^]^ Bone marrow mesenchymal stem cell (BMSCs)‐derived EVs can inhibit high phosphate‐induced vascular calcification through SIRT6‐HMGB1 deacetylation.^[^ ^45^ ^]^ In addition, drugs such as melatonin can alleviate vascular calcification by inducing VSMCs to release EVs with anti‐calcification activity.^[^ ^46^ ^]^ Jiang et al.^[^ ^19^ ^]^ found that SHep‐EVs could promote atherosclerosis by exacerbating the endothelial inflammatory response. Another study reported that SHep‐EVs aggravate atherosclerosis via the inhibition of cellular cholesterol efflux.^[^ ^29^ ^]^ However, it is unclear whether SHep‐EVs participate in vascular calcification.

We first analyzed the occurrence of vascular calcification in MAFLD patients using the diabetic coronary artery calcification cohort established previously by our research group. In this study, the prevalence of MAFLD in type 2 diabetes mellitus was 61.7%, which is consistent with the previously reported prevalence of 65.04%^[^ ^22^ ^]^ The odds of patients with fatty liver having coronary artery calcification (CACS > 0) were significantly higher than that of patients without fatty liver (66.0 vs. 39.2%), and hepatic steatosis is a risk factor for coronary artery calcification.

The pro‐vascular calcification effect of MAFLD has also been demonstrated in models of VitD overloading, natural aging, CKD, and atherosclerosis. To clarify whether EVs secreted by steatotic hepatocytes mediate the interaction between the liver and blood vessels, we constructed hepatocyte‐derived exosome tracer mice (AlbCre; CD63*^flox/flox^*) and found that hepatocyte‐derived EVs could reach the aorta and be taken up by VSMCs. In vitro, FFA‐treated mouse hepatocytes (AML‐12) and MAFLD patient plasma‐derived EVs showed a phenotype that promoted the osteogenic differentiation of VSMCs. By injecting SHep‐EVs into the ApoE^−/−^ mice by tail vein for 24 weeks, we found that SHep‐EVs significantly increased the atherosclerotic plaque area and intimal calcification.

Rab27a is a key molecule that mediates EV production and secretion.^[^ ^47^ ^]^ Rab27a was dramatically upregulated in the liver of HFD‐fed mice and FFA‐treated hepatocytes.^[^ ^33^ ^]^ Consistent with this report, we also found that Rab27a expression was significantly increased in the liver of HFD‐fed mice. To further clarify whether EVs derived from steatotic hepatocytes are key mediators of the pro‐atherosclerotic and calcifying effects of MAFLD, we constructed knockout mice exhibiting the liver‐specific deletion of Rab27a. Liver‐specific knockdown of Rab27a significantly reduced the number of exosomes in the mouse plasma and alleviated MAFLD‐associated vascular calcification and atherosclerosis. This evidence suggests that SHep‐EVs are important mediators of MAFLD‐related vasculopathy.

In ApoE^−/−^ and Rab27*^flox/flox^* mice, liver‐specific knockdown of Rab27a had different effects on blood lipids such that in Rab27*^flox/flox^* mice, blood lipid levels increased, particularly LDL‐c levels, whereas in ApoE^−/‐^ mice, blood lipid levels decreased. We analyzed the entire experimental process and, combined with the changes in blood lipid levels in liver‐specific knockdown of Rab27 mice (which also showed a decreasing trend; data not shown), we speculated that the possible reason for this discrepancy was the difference in the methods used to construct these two mouse models. When the Rab27*^flox/flox^* mice were injected with a vector driving liver‐specific Cre expression, the ApoE/hAATp promoter was used,^[^ ^48^ ^]^ whereas the TBG promoter was used in ApoE^−/−^ mice. The exogenous promoter may compete for the materials of the endogenous promoter during transcription and translation, thereby impairing the function of ApoE. It is well known that ApoE is a key protein involved in the regulation of lipid metabolism, particularly LDL‐c, so this may at least partially explain the observed result differences. Finally, the vascular media calcification was alleviated after the liver‐specific knockdown of Rab27a in Rab27^flox/flox^ mice. The main reason for this finding is that blood lipids have a limited role in VD‐induced media calcification. On the other hand, the reduction in hepatotoxic EV secretion after Rab27 knockdown may offset this adverse effect. Similar situations also occur in the context of SGLT2 inhibitor treatment. Studies have shown that SGLT2 has cardioprotective effects, but it also increases plasma LDL‐c levels.^[^ ^49^ ^]^ Currently, the promoters that can achieve liver‐specific knockdown mainly include the TBG, Alb, and APOE promoters. This effect should be avoided as much as possible in future experiments.

To explore the molecular mechanism by which SHep‐EVs promote atherosclerosis and calcification, we constructed an MAFLD mouse model, extracted primary hepatocytes, and conducted proteomic analyses of EVs derived from primary hepatocytes. In total, 144 differentially expressed proteins were identified, including 29 up‐regulated proteins and 115 down‐regulated proteins, among which Lgals3bp was the most strongly upregulated.

Lgals3bp (also known as Mac‐2 binding protein or 90K; encoded by *Lgals3bp*) is a secreted protein 585 amino acids in length,^[^ ^50^ ^]^ and its release in EVs is an important mode through which it is secreted. Lgals3bp can interact with the extracellular matrix, collagen, and four transmembrane proteins, in addition to playing a role in the progression of various tumors.^[^ ^41^, ^51^ ^]^ Lgals3bp is expressed in both normal and diseased aortic tissue and is present at higher levels in atherosclerotic plaques in addition to being upregulated in lipid‐loaded VSMCs.^[^ ^43^ ^]^ Lgals3bp levels were associated with all‐cause and cardiovascular mortality in a LURIC clinical cohort study that included 2,922 participants (62.7 ± 10.6 years, 62.7% male), and the underlying mechanisms may involve metabolic disorders and inflammatory stress.^[^ ^52^ ^]^ However, its clinical value and the molecular mechanisms involved in atherosclerosis and calcification warrant additional clinical prospective studies and efforts to explore the underlying mechanisms. In addition, previous studies have shown that Lgals3bp is up‐regulated in patients with MAFLD.^[^ ^53^ ^]^ More importantly, Lgals3bp can promote the osteogenic differentiation of stem cells by binding to Lgals3.^[^ ^42^ ^]^ This evidence suggests that Lgals3bp may be an important mediator through which SHep‐EVs can promote atherosclerosis and calcification. To test this hypothesis, we conducted in vivo and in vitro experiments which showed that in the liver of MAFLD model mice and FFA‐treated hepatocytes and EVs derived therefrom, Lgals3bp was significantly up‐regulated, while the knockdown of Lgals3bp alleviated MAFLD‐related arterial calcification and atherosclerosis. This suggests that Lgals3bp may be a key molecule involved in mediating the pro‐calcification effects of SHep‐EVs.

This study also has several limitations. SHep‐EVs may contain other functional proteins or nucleic acids that could be responsible for the pathology communication between the liver and the vascular system in addition to Lgals3bp. Second, the liver‐specific knockout shRNA constructs used in this study did not completely inhibit Rab27a and Lgals3bp expression in the liver. Despite these limitations, to our knowledge, this is the first report that MAFLD promotes vascular calcification and that SHep‐EVs can inducing osteogenic differentiation of VSMCs and promote the polarization of macrophages toward a pro‐inflammatory phenotype. In addition, we found that the expression of Lgals3bp was significantly up‐regulated in the liver tissue of MAFLD mice, and the liver‐specific knockdown of Lgals3bp reduced the levels of plasma TC and LDL‐c in C57 mice, indicating that Lgals3bp may become a therapeutic target in MAFLD and its extrahepatic complications. The current status of exosome research, including this work the focus is still on the effects of upregulated proteins or miRNA in exosomes on target cells/tissues. About the function of downregulation protein or miRNA should be investigated in the future.

## 4 Conclusion

In summary, we identified steatotic hepatocyte‐derived EVs as a novel mediator exacerbating MAFLD‐induced vascular calcification and atherosclerosis by transporting Lgals3bp to promote the osteogenic differentiation of VSMCs, induce foam cell formation, and facilitate the M1 polarization of macrophages. These results suggest that preventing abnormal hepatocyte EV production and blocking Lgals3bp biogenesis may be novel and effective therapeutic interventions that can block EV‐mediated pathologic communication between dysfunctional liver tissue and the vasculature, providing vascular protection against high‐fat injury.

## 5 Experimental Section

### Ethics Statement

The animal experiments were approved by the Animal Ethics Committee and followed the Guidelines for the Care and Use of Laboratory Animals at the University of South China (USC202205XS21). The collection and use of human blood samples and aorta were approved by The First Affiliated Hospital of the University of South China and the protocols followed were compliant with the ethical principles of the Helsinki Declaration (NCT04889053). Written informed consents were obtained from all human donors. No study participant received compensation.

### Human Samples

Serum samples from the volunteers were collected to isolate EVs (see Table S4, Supporting Information for details). The human aorta tissue in Figure 10J comes from the autopsy sample.

### Correlation Analysis Between Liver Fat Content and Coronary Artery Calcification

USCAC study **inclusion criteria**: Age ≥ 18 years old, Type 2 diabetes is diagnosed according to WHO diagnostic criteria, Low‐dose dual‐source CT coronary angiography could be performed at baseline investigation, those subjects can understand the purpose and procedure of this study and sign informed consent voluntarily. **Exclusion Criteria**: Those who have received coronary artery stenting or coronary artery bypass grafting, those with severe lung (respiratory failure), malignant tumor, mental illness or mental retardation, pregnant or lactating women or those with fertility planning, concomitant diseases (hyperparathyroidism, sarcoidosis, amyloidosis) affecting calcium balance and soft tissue calcification, contraindications of contrast agents, based on the judgment of the researcher, the compliance was poor and the study could not be completed according to the requirement.

### Measurement of CAC and Liver Fat Content

Coronary artery calcification (CAC) and liver fat content data acquisition methods and parameters were based on previous literature.^[^ ^54^ ^]^ In brief, all participants underwent the dual‐energy spectral CT scans on a 256‐slice CT scanner (Revolution GE Health Care). Each participant was in supine position, hands were raised over the head to avoid artifacts and was required to avoid movement and keep holding breath during the CT scans. Imaging protocols were as follows: tube voltage, 120 kVp; automatic tube current (range: 150–600 mA); gantry rotation time, 0.35 s; helical pitch, 1.375:1; detector coverage, 160 m; temporal resolution, 40 ms, prospective ECG‐gated axial scanning; reconstruction slice thickness, 2.5 mm, slice interval, 2.5 mm. All images were transmitted to a GE AW4.7 CT workstation for analysis.

### Animals

All animals were specific‐pathogen‐free mice and housed in the animal facility of the University of South China in controlled, identical temperature, humidity, and 12 h light‐dark cycle conditions. They had free access to sterilized water and the assigned diet.

### Alb^Cre^; Cd63^em(loxp‐mCherry‐loxp‐eGFP)3^ Mice

The generation and identification methods were reported in the previous work.^[^ ^21b^ ^]^ The primers for determining the insertion of loxP‐flanked mCherrygene were as follows: P1: 5′‐ CCTTCAGGGCTGCGTGGAGACTA‐3′; P2: TGCCCCTAAATATGCCTGCCTAAT; P3:5′‐AGTCCGCCCTGAGCAAAGA‐3′. If the loxP‐flanked mCherry was inserted, will amplify a 348‐bp product, whereas, in the absence of the insert, a 488‐bp product can be amplified. Cd63^em(loxp‐mCherry‐loxp‐eGFP)3^ mice were crossed with Alb Cre mice (Cyagen Biosciences Inc. Guangzhou, China) to obtain Cre; Cd63^em(loxp‐mCherry‐loxp‐eGFP)3^ mice. The abdominal aortas of these three types of mice were harvested and processed for the detection of cherry and eGFP signals with a fluorescence microscope. The primers for the AlbCre transgene were as below: Alb‐Cre‐F(F1): 5’‐GAAGCAGAAGCTTAGGAAGATGG‐3’; Alb‐Cre‐R(R1):5’‐TTGGCCCCTTACCATAACTG‐3’. Primers for Wild type PCR: WT‐F (F2): 5’‐TGCAAACATCACATGCACAC‐3’; Alb‐Cre‐R (R1): 5’‐TTGGCCCCTTACCATAACTG‐3’. A 390‐bp PCR product would be generated in mice homozygous for the Alb Cre allele and a 351‐bp PCR product would be yielded in the wild‐type mice.

### Hepatic EVs Secretion Blocking Mice

Rab27a is a key protein that mediates the secretion of EVs.^[^ ^32^ ^]^ Rab27a*^flox/flox^* mice were constructed and combined them with liver‐specific Cre expression adeno‐associated virus (pAAV‐ApoE/hAATp‐MCS‐EGFP‐3Flag‐SV40 PolyA) to achieve hepatocytes‐specific knockout of Rab27a, which blocks the secretion of liver EVs specifically.

### Hepatocytes Lgals3bp‐Specific Knockout Mice

Hepatocytes‐specific knockout of Lgals3bp by using a liver‐specific knockout adeno‐associated virus (TBGp‐EGFP‐MIR155(MCS)‐SV40 PolyA), the sequence is *AGGAGCTGTTCGAGCTGCAGTTCAA*.

### Animal Model of Vascular Calcification—Vascular Calcification Model of Vitamin D Overload

The vitamin D overload model is a widely used and reproducible model of medial calcification.^[^ ^21^ ^]^ C57BL/6 wild‐type mice a chow diet or high‐fat diet (R&D 12492) was given, followed by vitamin D (daily injection of 750 U g^−1^ body weight for 4 consecutive days), continued feeding for 7 days, and were then taken for analysis.

### Animal Model of Vascular Calcification—Chronic Kidney Disease Vascular Calcification Model

Eight‐week‐old female DBA/2 mice were purchased from Vital River Laboratory Animal Technology Co., Ltd. and maintained in a specific pathogen‐free environment. DBA/2 mice were highly susceptible to ectopic calcification, in contrast to C57BL/6. Female mice were used because they showed higher susceptibility to calcification than male mice.^[^ ^55^ ^]^ Modified AIN‐76A purified rodent diet with 1.8% P and 0.2% adenine(Ctrl) and high fat (60% FDC) purified rodent diet with 0.2% Adenine, 1.8% phosphorus(HFD) were purchased from Dyets Inc.

### Animal Model of Vascular Calcification—Atherosclerotic Vascular Calcification Model

For the diet‐induced atherosclerotic calcification model, eight‐week‐old male ApoE^−/−^were randomly provided with a regular chow diet for 24 weeks as the control group, or a diet that had a nutritional profile similar to the western diet (D12108C, Research Diets) for 24 weeks as the experimental group. To assess the effects of EVs on atherosclerotic vascular calcification, 8‐week‐ApoE^−/−^ male mice were treated with 75 µg of SHep‐EVs, or an equal volume of solvent (PBS; 100 µL) by tail vein injection one time a week for 24 weeks. The mice were fasted for another 12 h before euthanasia to assess blood lipids and other biochemical indicators. Blood was collected, and the whole aortas, including the brachiocephalic and femoral arteries, were removed for further analysis.

### Cell Culture—Primary VSMCs Isolation and Culture

VSMCs were isolated from the 3‐4‐week‐old C57BL/6 mice. Briefly, mice were anesthetized using intraperitoneal injection of pentobarbital (1%, 50 mg kg^−1^); mice were then soaked using 75% alcohol for 30 s, followed by opening of the thoracic cavity, perfusing with pre‐chilled PBS to remove visceral blood, clipping of lung, esophagus and perivascular adipose tissue, and subsequent isolation of the aorta; the isolated aortas were placed in pre chilled F12 basal medium to remove perivascular residual adipose tissue again, and the aortas were subsequently placed in a compound digestive enzyme (Collagenase type II, elastase, trypsin inhibitor(Worthington)) to digest for 10–15 min, the aortic adventitia was removed intact using microscissors and the aortas were subsequently clipped off using microscissors to scrape the aortic intima; aortas from which the inner and outer membranes were removed were again placed in a new compound digestive enzyme, and digestion was continued for ≈2 h until the aortas detached into a cell suspension; subsequently the cell suspension be centrifugation at 300 g for10 min then plating into 60 mm dishes (2 mice) using a complete medium containing 20% FBS, 1% P/S and 1% smooth muscle cell growth factor(Shanghai Zhong Qiao Xin Zhou Biotechnology Co., Ltd. Cat. 1152). Subsequent experiments were then performed using smooth muscle cells from generations 2–6 and identified by immunofluorescence detection of the smooth muscle marker a‐SMA.

### Cell Culture—Primary Hepatocyte Isolation and Culture

This protocol for primary hepatocyte extraction involves the following steps: 1) Cleaning the work area and priming pump; 2) Flushing the pipeline with 75% ethanol for 10 min; 3) Flushing the pipeline with PBS for 10 min to remove residual ethanol; 4) Anesthetizing the mice; 5) Exposing the hepatic portal vein and inferior vena cava; 6) Inserting a perfusion needle into the inferior vena cava at the fork of the right renal vein; 7) Perfusing preheated HBSS (37 °C) at the rate of 4 ml min^−1^ and cutting the portal vein (dosage: 35 mL per mouse); 8) Adding type IV collagenase (0.5 mg mL^−1^, prepared by HBSS) to the container when the HBSS was about to run out, and continuing the perfusion for ≈10 min followed by removal of the liver and gallbladder; 9) Placing the liver into the culture medium and shaking it to free the hepatocytes; 10) Blowing 3 times with a pasteurized straw and filtering through a 75 µm cell filter; 11) Centrifuging at 4 °C for 5 min at 50 g, removing the supernatant, and repeating this step twice; 12) Inoculating the cell precipitation into the culture medium and changing the solution after 4 h. Immunofluorescence and western blot were used to detect albumin to identify the extracted hepatocytes.

### Cell Culture—Mouse Hepatic Cell Lines (AML12)

AML‐12 cell lines were obtained from the Procell Life Science & Technology Co., Ltd. They were cultured in DMEM/F12 with 10% fetal bovine serum (FBS) (Sigma), 40 ng ml^−1^ dexamethasone (Sigma), 0.45% Liquid Media Supplement (ITS, Sigma) and incubated in a humidified atmosphere at 37 °C and 5% CO_2_.

### SHep‐EVs with Lgals3bp Knockdown

AML‐12 transfected with shLgals3bp showing the highest silencing efficiency or with shCtrl were selected to produce EVs for further experiments. The siRNA sequences were as follows: Sh1#: 5’‐CGAAGAATCGAGGTCAGCATGTCTT‐3’; Sh2#: 5’‐AGGAGCTGTTCGAGCTGCAGTTCAA‐3’; Sh3#: 5’‐ TATGGCTCAGTAGCCCGGTACAATA‐3’; Sh‐Ctrl#: 5’‐ TTCTCCGAACGTGTCACGTAA‐3’. Forty‐eight hours after adenovirus transfection, the medium was replaced with DMEM/F12 medium containing 10% exosomes‐free FBS and 500 µm FFA and continued to be cultured for 24 h. EVs in the medium were collected for subsequent experiments.

### Lipid‐Accumulating Hepatocyte Model

AML‐12 hepatocytes were chronically exposed to different concentrations of saturated FFA (oleic acid: palmitic acid V/V = 2:1) from 0–600 µm for 24 h,^[^ ^28^ ^]^ the effect of lipid loading on hepatocytes was measured by ORO staining.

### Human Aortic Smooth Muscle Cells

hVSMC (ZQY016; Zhong Qiao Xin Zhou Biotechnology Co.,Ltd., Shanghai, China) were incubated in DMEM/F12 (11330057; Gibco) + 10% FBS + 1% P/S.

### Osteogenic Differentiation

To induce osteogenic differentiation of VSMCs, cells were cultured in osteogenic differentiation medium (OM) with the following additions for a total of 7–21 days: 10 nmol L^−1^ dexamethasone, 10 mmol L^−1^ β‐glycerol phosphate, and 100 µmol L^−1^ L‐ascorbic acid 2‐phosphate (termed osteogenic media, OM). The expression levels of α‐SMA and RUNX2 were assessed at 7 days after induction. ALP activity and stain were performed at 7 days after induction, and alizarin red staining was performed 21 days after induction.

### Atherosclerosis Analysis

The experimental mice were perfused by cardiac puncture with 4% (w/v) paraformaldehyde to wash out blood from the heart and all vessels after euthanasia. After removing the surrounding fat and connective tissues, the entire aorta was examined under a stereomicroscope. The whole aorta was excised from the aortic arch to the common iliac artery and stained with ORO staining. To analyze the atherosclerotic lesions, cross sections of the aortic root were stained with H&E, ORO and Masson's reagent, respectively. Fluorescence images were obtained by the ZEISS LSM880 confocal microscope and quantified using IMAGEPRO PLUS software.

### Calcification Analysis—Whole Alizarin Red Staining

Images were collected after 24 h staining with 3% ARS containing 1% KOH.

### Calcification Analysis—Aorta Section Alizarin Red Staining

Sections of 5 µm thickness were prepared for paraffin or OCT‐embedded samples and stained with Alizarin Red S solution for 5 min. Subsequently, the sections were washed twice with tap water and sealed with neutral gum.

### Calcification Analysis—Aortic Calcium Content Assay

The whole aortas were decalcified with 5% HCl at 4 °C for 48 h. After testing the protein concentration, the calcium content in the supernatant was assessed using a commercial kit from Beyotime Biotechnology (S1063S). The vascular calcium content was normalized to the concentration of protein.

### Calcification Analysis—Aorta Osteosense Analysis

OsteoSense 680 (Perkin Elmer) was a bisphosphonate‐derivatized near‐infrared fluorescent imaging agent that binds to hydroxyapatite. OsteoSense 680 marks early osteogenic activity in the vasculature and is commonly used to detect arterial microcalcification.^[^ ^31b^ ^]^ Calcification in the mouse aorta was analyzed via Osteosense 680 (100 µL) tail vein injections 24 h prior to euthanization and imaging. Aortas were perfused with saline, dissected, and imaged using fluorescent reflection imaging (PerkinElmer IVIS Spectrum). Imaging was performed using excitation/emission filter sets of 650 nm/700 nm.

### VSMC Calcification Analysis—Alizarin Red Staining

Matrix calcium deposition was analyzed by the use of Alizarin Red staining. Briefly, cells were washed in PBS then fixed in 4% paraformaldehyde for 30 min, and then washed with distilled water. Cells were stained with freshly prepared and filtered Alizarin Red S (Sigma), pH 4.2, with excess stain being removed with four subsequent distilled water washes. Images were taken using a Zeiss camera.

### ALP Staining

Briefly, cells were washed in PBS then fixed in 4% paraformaldehyde for 10 min, and then washed with distilled water. Cells were stained with freshly prepared ALP staining working solution (according to the manufacturer's instructions, Beyotime Biotechnology, C3206).

### ALP Activity Assay

Tissue non‐specific alkaline phosphatase (TNAP) activity was analyzed using whole cell lysates and the TNAP enzyme activity assay (Beyotime Biotechnology, P0321S).

### qRT‐PCR

Total RNA was extracted with an Ultrapure RNA kit (CW0581M; CWBIO, China) and subjected to cDNA synthesis with the NovoScript®Plus All‐in‐one 1st Strand cDNA Synthesis SuperMix (E047; Novoprotein, China). Then, qRT‐PCR was performed with 2x SYBR Green qPCR Master Mix (B21202; Selleck, China) on the FTC‐3000 V1.0.3.44 real‐time PCR system. GAPDH served as the reference genes for normalization. The primers for qPCR are listed in Table S5 (Supporting Information).

### Immunohistochemistry

The expression of RUNX2 (1:200) and Rab27a (1:200) in the vascular or liver tissue were examined by immunohistochemistry. Briefly, the tissue sample was fixed in 4% paraformaldehyde for 48 h, then embedded in paraffin and cut into 5 µm sections. Subsequently, sections were incubated in the oven at 65 °C for 2 h, followed by dewaxing in xylene three times at 20 min intervals and then dehydrating in 99%, 95%, and 75% ethanol for 5 min. Citrate was used for antigen retrieval at boiling temperature for 20 min, and hydrogen peroxide incubation was performed at room temperature in a dark box for 10 min to diminish endogenous peroxidases. Sections were then blocked by sheep serum for 30 min, followed by incubating with a specific primary antibody overnight at 4 °C. The next day, sections were rewarmed at room temperature for 30 min and then incubated with a secondary antibody for 1 h at room temperature. A DAB kit was used to detect positive staining. Finally, sections were counterstained with hematoxylin.

### Immunofluorescence

Frozen sections (5 µm) were used for immunofluorescence detection. Sections were incubated with Triton X‐100 for 20 min (for nucleoprotein), and subsequently blocked by 5% BSA for an hour in a humidified chamber to block non‐specific binding. Slides were incubated with antibodies against RUNX2, α‐SMA, CD68, or CD86 at 4 °C overnight and then exposed to fluorescent label conjugated secondary antibodies (Alexa Fluor, Invitrogen). Nuclei were counter‐stained using DAPI (the details about agents and antibodies refer to Table S6, Supporting Information). Images were captured using a Zeiss microscope and were quantified using Image‐pro Plus. Results were expressed as the ratio of relative fluorescence intensity compared with the controls. All quantifications were performed by two observers without knowledge of the identities of the samples.

### Western Blot

Protein extracts were obtained by lysing the cells/tissue in RIPA buffer (Cowin Bio., CW2333) supplemented with protease inhibitor cocktail (Cowin Bio, CW2200, USA). The lysates were incubated on ice for 30 min and then centrifuged for 15 min at 12000 rpm. The protein supernatant was carefully collected, and the protein content was measured by Bradford assay. The samples were mixed in 5× loading buffer and denatured at 95 °C for 10 min. Twenty micrograms of protein was loaded onto 10% SDS‐PAGE together with the protein marker (Thermo, #26616). The resolved proteins were transferred to PVDF (Millipore, USA) in the Bio‐Rad Trans‐Blot cell with wire electrodes according to the manufacturer's instructions. The membranes were blocked in 5% skim milk dissolved in 1× TBST buffer at room temperature for 2 h, followed by incubation with the primary antibody at 4 °C overnight. The membranes were washed three times in TBST, and then incubated with corresponding secondary antibodies conjugated with horseradish peroxidase at a dilution of 1:3000 for 1 h at room temperature. Finally, the membranes were washed another three times in TBST and visualized by western blotting detection kit (Advansta, USA). Protein bands were visualized by films in the darkroom or using the Bio‐Rad ChemiDoc Imaging System.

### Isolation and Characterization of EVs

The hepatocytes were cultured in the medium containing exosomes‐depleted serum. After treatment, the medium was collected and centrifuged at 2000 g for 15 min at 4 °C and centrifuged again at 12 000 g for 30 min at 4 °C. Then, the supernatants were passed through a 0.22 µm filter (Millipore) and ultracentrifuged at 100 000 g for 90 min at 4 °C. The pellets were washed with PBS followed by a second ultracentrifugation at 110 000 g for 90 min at 4 °C and resuspended in PBS. The BCA Protein Assay Kit (Elabscience) or the ZetaView® nanoparticle tracking analyzer (Particle Metrix, Meerbusch, Germany) was used to measure the exosomes concentration.

### EVs Tracing

To label exosomes, 1,1′‐dioctadecyl‐3,3,3′,3′tetramethylindotricarbocyanine iodide (DiR; D12731, Thermo Fisher Scientific/Invitrogen), diluted in ethanol at a concentration of 1 mg mL^−1^, was mixed with exosomes at the ratio of 2 µg DiR/100 µg exosomes in PBS for 1 h followed by ultracentrifugation at a speed of 100 000 g for 1 h to remove unincorporated DiR and ethanol. The precipitate was resuspended in PBS at a concentration of 0.75 µg exosome/1 µL PBS. PBS vehicle or DiR‐labelled exosomes were injected via the tail vein, and in vivo fluorescence images were captured with a PerkinElmer IVIS Spectrum after 6 and 24 h and analyzed by in vivo imaging software.

### Exosomes Labelling

Purified exosomes samples were dyed with 3,3' – Dioctadecyloxacarbocyanine Perchlorate (DiO, Green, Absin, China) and incubated for 20 min at 37 °C. Next, The VSMCs were incubated with DiO‐labelled exosomes or PBS for 4 or 12 h and then dyed with phalloidin (Sigma, Darmstadt, Germany) and DAPI (Sigma). Labeled cells were imaged by confocal microscopy.

### Statistical Analysis

Statistical analyses were performed using GraphPad Prism 9.0. All data were tested for normality and equal variance. If passed, Student's t test was used to compare two groups or one‐way analysis of variance (ANOVA) followed by Tukey post hoc test for comparisons among >2 groups. Otherwise, nonparametric tests (Mann‐Whitney U test or Kruskal‐Wallis test followed by Dunn's post hoc test) were used. *P* value <0.05 was considered statistically significant.

## Conflict of Interest

The authors declare no conflict of interest.

## Author Contributions

J.‐H.L., H.X., X.‐Y.Z., and Z.‐L.Z. conceptualized the study. Z.‐L.Z., Q.Y., S.‐Q.Y., S.‐Y.Z., Z.‐B.Z., Q.C., G.‐Q.Z., and A.‐Q.L. performed experiments. Z.‐L.Z., J.‐H.L., H.X., and X.‐H.X. analyzed and interpreted the data. G.L. and H.L. were responsible for the interpretation and analysis of the coronary artery calcium score and liver fat content data. J.‐Y.L. responsible for statistical analysis of clinical data. Z.‐L.Z. wrote the manuscript, Z.‐X.W., and J.‐H.L. revised the manuscript.

## Supporting information



Supporting Information

## Data Availability

The data that support the findings of this study are available from the corresponding author upon reasonable request.
