# Supplementary material for: Hepatic Steatosis Aggravates Vascular Calcification via Extracellular Vesicle‐Mediated Osteochondrogenic Switch of Vascular Smooth Muscle Cells
Source: Adv Sci (Weinh). 2024 Dec 16;12(5):2408660. doi: 10.1002/advs.202408660 (PMC11791995; doi:10.1002/advs.202408660)
Supplement: Supplementary file 1 — Supporting Information [file ADVS-12-2408660-s001.docx]

Supporting Information

**Hepatic Steatosis Aggravates Vascular Calcification via Extracellular Vesicle-mediated Osteochondrogenic Switch of VSMCs**

*Zhao-Lin Zeng, Zhi-Bo Zhao, Qing Yuan, Shi-Qi Yang, Zhen-Xing Wang, Zuo Wang, Shi-Yu Zeng, An-Qi Li, Qian-Chen, Guo-Qiang Zhu, Xin-Hua Xiao, Guang-Hua Luo*, *Hai-Yan Luo*, *Jiao-Yang Li, Xu-Yu Zu, Hui Xie, Jiang-Hua Liu*

**Contains 7 tables and 16 figures.**

**Table S1. Characteristics of the Study Participants for analyzing the influence of fatty liver on coronary artery calcification.**

| Characteristics | Overall(N=520) | Non-MAFLD(n=199) | MAFLD(n=321) | *P* value |
| --- | --- | --- | --- | --- |
| Age (years) | 54.6±9.8 | 55.1±9.9 | 54.2±9.8 | 0.33 |
| Male sex (%) | 311(59.8) | 115(57.8) | 196(61.1) | 0.16 |
| Diabetes duration (years) | 5(1-10) | 6(1-10) | 5(1-10) | 0.36 |
| HbA1c | 10(8.2-11.82) | 10.2(8.15-11.85) | 9.9(8.2-11.84) | 0.53 |
| HR (bpm) | 89(79-98) | 90(79-98) | 88(79-97) | 0.28 |
| SBP (mmHg) | 132.4±19.9 | 132.0±20.4 | 132.7±19.7 | 0.67 |
| DBP (mmHg) | 84.9±11.3 | 85.1±11.2 | 84.7±11.4 | 0.37 |
| BMI (kg/m^2^) | 23.79(21.54-26.12) | 23.03(21.33-25.07) | 24.23(21.91-26.9) | *P*＜0.01 |
| TC (mmol/L) | 4.56(3.86-5.25) | 4.53(3.87-5.2) | 4.59(3.79-5.3) | 0.81 |
| TG (mmol/L) | 1.67(1.15-2.7) | 1.62(1.08-2.55) | 1.71(1.17-2.75) | 0.18 |
| HDL (mmol/L) | 1.02(0.85-1.26) | 1.02(0.88-1.26) | 1.02(0.82-1.27) | 0.35 |
| LDL (mmol/L) | 2.68(2.16-3.29) | 2.69(2.15-3.29) | 2.67(2.18-3.29) | 0.91 |
| Glu (mmol/L) | 11.82(7.9-18.08) | 10.93(7.33-19.06) | 11.98(8.09-17.74) | 0.62 |
| ALT (U/L) | 18(12.35-26.2) | 17.8(11.8-25.4) | 18.15(12.58-26.83) | 0.44 |
| AST (U/L) | 16.9(13.7-22.7) | 16.5(13.3-22.15) | 17.5(13.9-23.3) | 0.24 |
| ALP (U/L) | 76(61-97) | 76(59-94) | 76(62-99) | 0.28 |
| Calcium (mmol/L) | 2.26±0.14 | 2.25±0.13 | 2.26±0.14 | 0.58 |
| Phosphorus (mmol/L) | 1.05(0.93-1.19) | 1.05(0.93-1.19) | 1.05(0.93-1.19) | 0.88 |
| PTH (mmol/L) | 40.55(28.83-73.06) | 42.39(27.44-90.3) | 39.89(29.13-62.87) | 0.11 |
| 25(OH)D(mmol/L) | 19(13.86-25.08) | 19.93(14.71-27) | 18.3(13.3-23.7) | 0.02 |
| CAC(%) | 55.8% | 39.2% | 66.0% | *P*＜0.01 |

Data are presented as the mean±SD or median (25th–75th quartiles).

**HbA1c:** Hemoglobin A1c; **HR:** Heart rate; **SBP:** Systolic blood pressure; **DBP:** Diastolic blood pressure; **BMI:** Body mass index; **TC:** Total cholesterol; **TG:** Triglycerides; **HDL:** High-density lipoprotein; **LDL:** Low-density lipoprotein; **Glu:** Glucose; **ALT:** Alanine transaminase; **AST:** Aspartate transaminase; **ALP:** Alkaline phosphatase; **PTH:** Parathyroid hormone; **CAC:** Coronary artery calcification, coronary artery calcium score>0.

**Table S2. Correlation analysis between different factors and coronary artery calcification**

| Independent Variables | Standardized coefficient | t | P value |
| --- | --- | --- | --- |
|  | Beta |  |  |
| Gender | -0.005 | -0.128 | 0.898 |
| HbA1c | -0.003 | -0.07 | 0.944 |
| SBP | -0.016 | -0.392 | 0.695 |
| BMI | 0.015 | 0.356 | 0.722 |
| Diabetes duration | 0.22 | 4.93 | <0.001 |
| Glu | 0.018 | 0.423 | 0.672 |
| TC | -0.068 | -1.606 | 0.109 |
| Age | 0.138 | 3.092 | 0.002 |
| Fatty liver | 0.17 | 4.031 | <0.001 |

**Table S3. Multiple logistic regression model to analyze the effect of fatty liver on vascular calcification**

| Independent Variables | OR | 95% Confidence Interval | | P value |
| --- | --- | --- | --- | --- |
|  |  | Lower | Upper |  |
| Gender | 1.219 | 0.802 | 1.853 | 0.355 |
| Age | 1.069 | 1.043 | 1.096 | <0.001 |
| HbA1c | 1.005 | 0.927 | 1.090 | 0.897 |
| SBP | 0.999 | 0.988 | 1.009 | 0.791 |
| BMI | 1.076 | 1.010 | 1.146 | 0.024 |
| Diabetes duration | 1.079 | 1.040 | 1.119 | <0.001 |
| Glu | 0.993 | 0.969 | 1.017 | 0.566 |
| TC | 1.048 | 0.886 | 1.239 | 0.587 |
| Fatty liver | 1.414 | 1.280 | 1.564 | <0.001 |

**Table S4. Characteristics of donors of plasma extracellular vesicles.**

| Characteristics | Non-MAFLD(n=9) | MAFLD(n=9) | *P* value |
| --- | --- | --- | --- |
| Age (years) | 22(22-23) | 23(22-39) | 0.43 |
| Male sex (%) | 8(88.9%) | 8(88.9%) | 0.15 |
| Height(cm) | 168.00±4.72 | 172.6±7.45 | 0.14 |
| Weight (kg) | 57.33±11.67 | 84.44±16.04 | *P*＜0.01 |
| SBP (mmHg) | 118.4±11.46 | 129.4±17.36 | 0.13 |
| DBP (mmHg) | 72.56±8.88 | 75.89±7.59 | 0.41 |
| BMI (kg/m^2^) | 20.24±3.26 | 28.18±3.49 | *P*＜0.01 |
| ALT (U/L) | 13.60(10.55-24.55) | 33.2(15.35-77.7) | *P*＜0.05 |
| AST (U/L) | 15.3(13.15-19.80) | 24.20(16.35-37.55) | 0.10 |
| Albumin | 49.93±2.14 | 50.49±3.47 | 0.69 |
| TC (mmol/L) | 3.88±0.92 | 4.76±0.94 | 0.06 |
| TG (mmol/L) | 1.01±0.28 | 2.41±1.07 | *P*＜0.01 |
| HDL (mmol/L) | 1.34±0.20 | 0.97±0.20 | *P*＜0.01 |
| LDL (mmol/L) | 2.23±0.84 | 3.05±0.75 | *P*＜0.05 |
| BUN(mmol/L) | 4.04±0.63 | 4.29±0.75 | 0.46 |
| Scr(μmol/L) | 81.67±9.50 | 84.64±15.97 | 0.64 |

Data are presented as the mean±SD or median (25th–75th quartiles).

**SBP:** Systolic blood pressure; **DBP:** Diastolic blood pressure; **BMI:** Body mass index; **TC:** Total cholesterol; **TG:** Triglycerides; **HDL:** High-density lipoprotein; **LDL:** Low-density lipoprotein; **ALT:** Alanine transaminase; **AST:** Aspartate transaminase; **ALP:** Alkaline phosphatase; **PTH:** Parathyroid hormone; **BUN:** Blood Urea Nitrogen; **Scr:** serum creatinine.

| **Table S5. Primers for qPCR.** | |
| --- | --- |
| **Gene** | **Sequence** |
| mαSMA-F | GTCCCAGACATCAGGGAGTAA |
| mαSMA-R | TCGGATACTTCAGCGTCAGGA |
| mSM22α-F | GTGTGATTCTGAGCAAATTGGTG |
| mSM22α-R | ACTGCTGCCATATCCTTACCTT |
| mRunx2-F | ATGCTTCATTCGCCTCACAAA |
| mRunx2-R | GCACTCACTGACTCGGTTGG |
| mGAPDH-F | AGGTCGGTGTGAACGGATTTG |
| mGAPDH-R | TGTAGACCATGTAGTTGAGGTCA |
| mLgals3bp-F | TGCTGGTTCCAGGGACTCAA |
| mLgals3bp-R | CCACCGGCCTCTGTAGAAGA |
| mRab27a-F | TCGGATGGAGATTACGATTACCT |
| mRab27a-R | TTTTCCCTGAAATCAATGCCCA |
| hGAPDH-F | ACAACTTTGGTATCGTGGAAGG |
| hGAPDH-R | GCCATCACGCCACAGTTTC |
| hSM22a-F | CCGTGGAGATCCCAACTGG |
| hSM22a-R | CCATCTGAAGGCCAATGACAT |
| hRUNX2-F | TCTCAGATCGTTGAACCTTGCTA |
| hRUNX2-R | TGGTTACTGTCATGGCGGGTA |
| hLgals3bp-F | GTTGCTGGCCCGGTACAAA |
| hLgals3bp-R | CCAGGAACTGTCTGTCACAAAG |

**F:** forward primer; **R:** reverse primer; **m:** mouse; **h:** human.

**Table S6. The main primary antibodies used in this study.**

| **Primary antibodies** | **Company** | **Cat.** |
| --- | --- | --- |
| RUNX2 | Cell signaling technology | 12556 |
| α-SMA | Cell signaling technology | 69319 |
| CD68 | Servicebio | GB113109 |
| iNOS | Proteintech | 22226-1-AP |
| Lgals3bp(R) | Proteintech | 10281-1-AP |
| Lgals3bp(M) | Proteintech | 60066-1-LG |
| Rab27a | Abcam | ab55667 |
| β-actin | Proteintech | 81115-1-RR |
| GAPDH | Proteintech | 10494-1-AP |
| Albumin | Proteintech | 16475-1-AP |

**Table S7. The main reagents used in this study.**

| **Agents** | **Company** | **Cat.** |
| --- | --- | --- |
| DiO dye | Absin | abs45153674 |
| Plasma Exosome Isolation Reagent | Thermo Fisher | 4484451 |
| GW4869 | Sigma-Aldrich | D1692 |
| OsteoSense 680 | Perkin Elmer | NEV10020EX |
| Calcium content detection kit | Beyotime | S1063S |
| Ox-LDL(Human) | Yiyuan biotechnology | YB-002 |
| IFN-γ | Solarbio | P00106 |
| LPS | Solarbio | L8880 |
| IL-1β ELISA kit | Elabscience | E-EL-M0037C |
| LGALS3BP ELISA kit | Elabscience | E-EL-H1456C |
| Ultrapure RNA extraction kit | CWBIO | CW0581M |
| Protein Maker | Thermo Fisher | 26616 |
| BCA protein concentration assay kit | Elabscience | E-BC-K318-M |
| DMEM/F-12 medium | Gbcio | 12400024 |
| DNA Maker | Takaba | 3591A |
| Dexamethasone | Sigma-Aldrich | D4902 |
| 2-Phospho-L-ascorbic acid trisodium salt | Sigma-Aldrich | 49752 |
| β-glycerophosphate | Sigma-Aldrich | 50020 |
| Palmitic acid | Sigma-Aldrich | 57-10-3 |
| Oleic acid | Sigma-Aldrich | 112-80-1 |
| Alizarin Red S | Sigma-Aldrich | 130-22-3 |


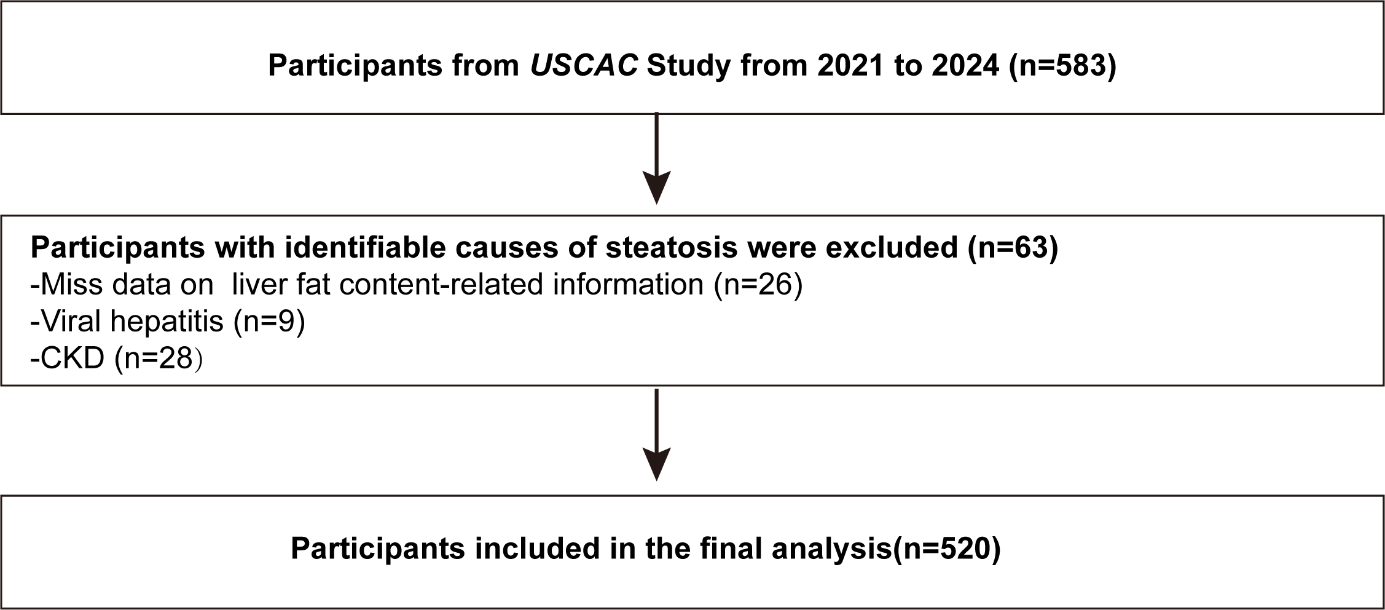


**Figure S1. Flow chart of study participants.**

USCAC: Clinical Research of Coronary Artery Calcification in Type 2 Diabetes Mellitus (NCT04889053); CKD: chronic kidney disease.


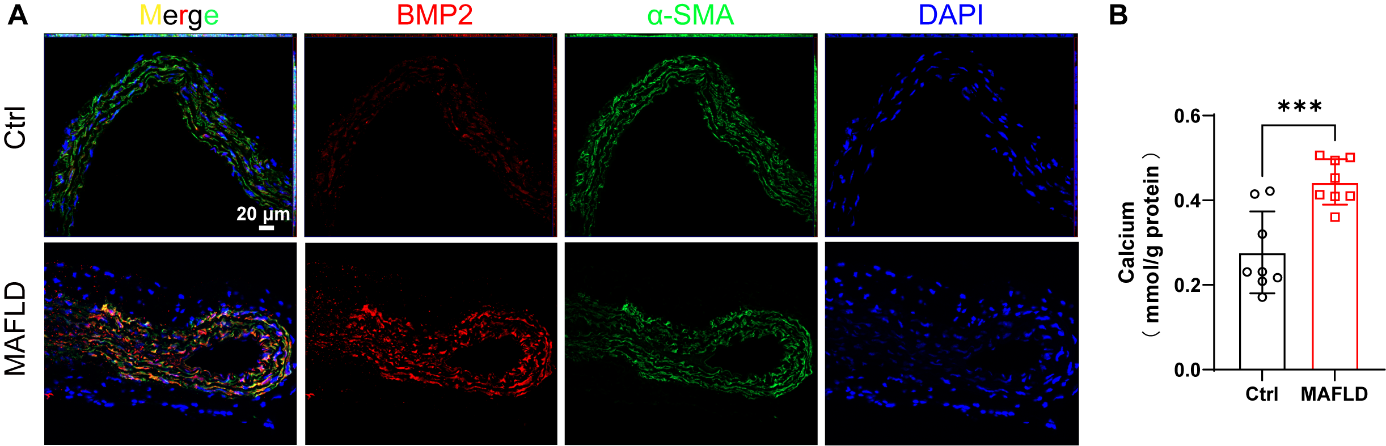


**Figure S2. MAFLD promotes arterial calcification.**

**A.** Immunofluorescence analysis of BMP2 and α-SMA protein expression in the aorta of 15-month-old C57 male mice fed a regular diet (Ctrl) or a high-fat diet (MAFLD) for 12 weeks, scale bar=20 μm; **B.** Aortic calcium assay (n=8). Data are presented as mean±SD, *p* values were determined by unpaired two-tailed Student’s t-test, ****p*＜0.001.


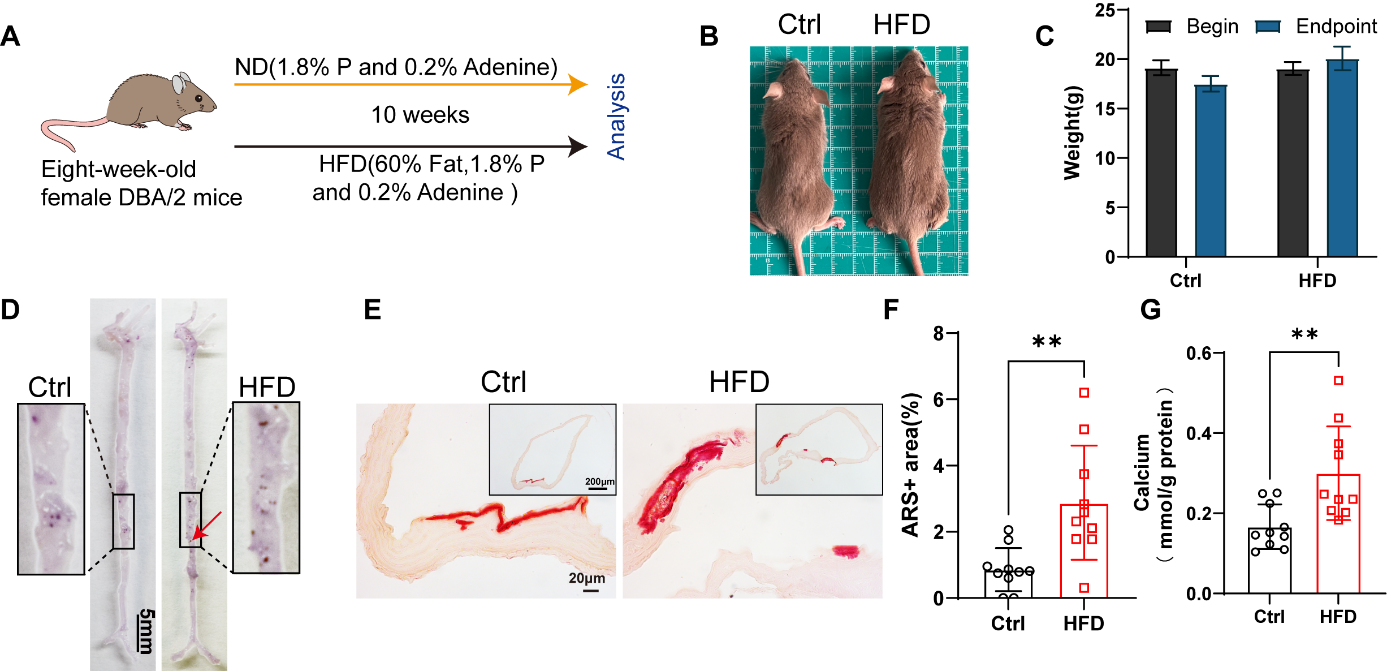


**Figure S3. MAFLD exacerbates arterial calcification in uremia-related vascular calcification in DBA/2 mice.**

**A.** Schematic diagram of the animal experiment; **B.** Mouse size comparison; **C.** Weight Analysis; **D.** ARS staining of the whole aorta, scale bar=5 mm; **E, F.** ARS staining and statistics of aortic sections, scale bar=200 or 20μm; **G.** Aortic Calcium Assay(n=10). Data are presented as mean±SD, *p* values were determined by unpaired two-tailed Student’s t-test, ***p*＜0.01.


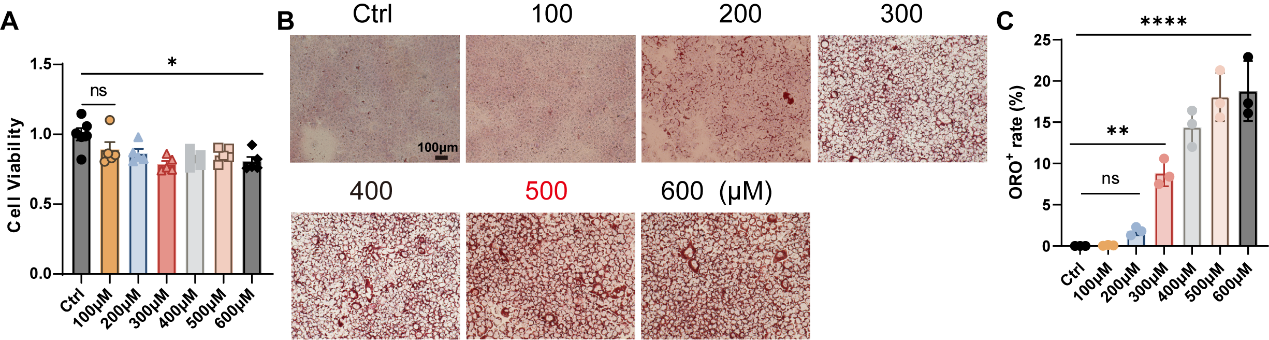


**Figure S4. Model construction of steatotic hepatocytes.**

**A.** Cell viability assay of AML-12 mouse hepatocytes after treatment with different concentrations of FFAs for 24 h (n=5), data are presented as mean±SD, *p* values were determined by one-way ANOVA followed by Tukey’s test, 100 μM *vs.* Ctrl group "ns" represents no significant difference, and 200-600 μM of FFAs *vs.* Ctrl group "*" represents *p* < 0.05; **B.** Representative images of ORO staining of AML-12 mouse hepatocytes treated with different concentrations of FFAs for 24h, scale bar=100 μm; **C.** Statistics of ORO staining positive rate (n=3). data are presented as mean±SD, *p* values were determined by one-way ANOVA followed by Tukey’s test, 100 μM and 200 μM FFAs *vs.* Ctrl group "ns" represents no significant difference, and 300 μM of FFAs *vs.* Ctrl group "**" represents *p* < 0.01, 400-600 μM of FFAs *vs.* Ctrl group "****" represents *p* < 0.0001.


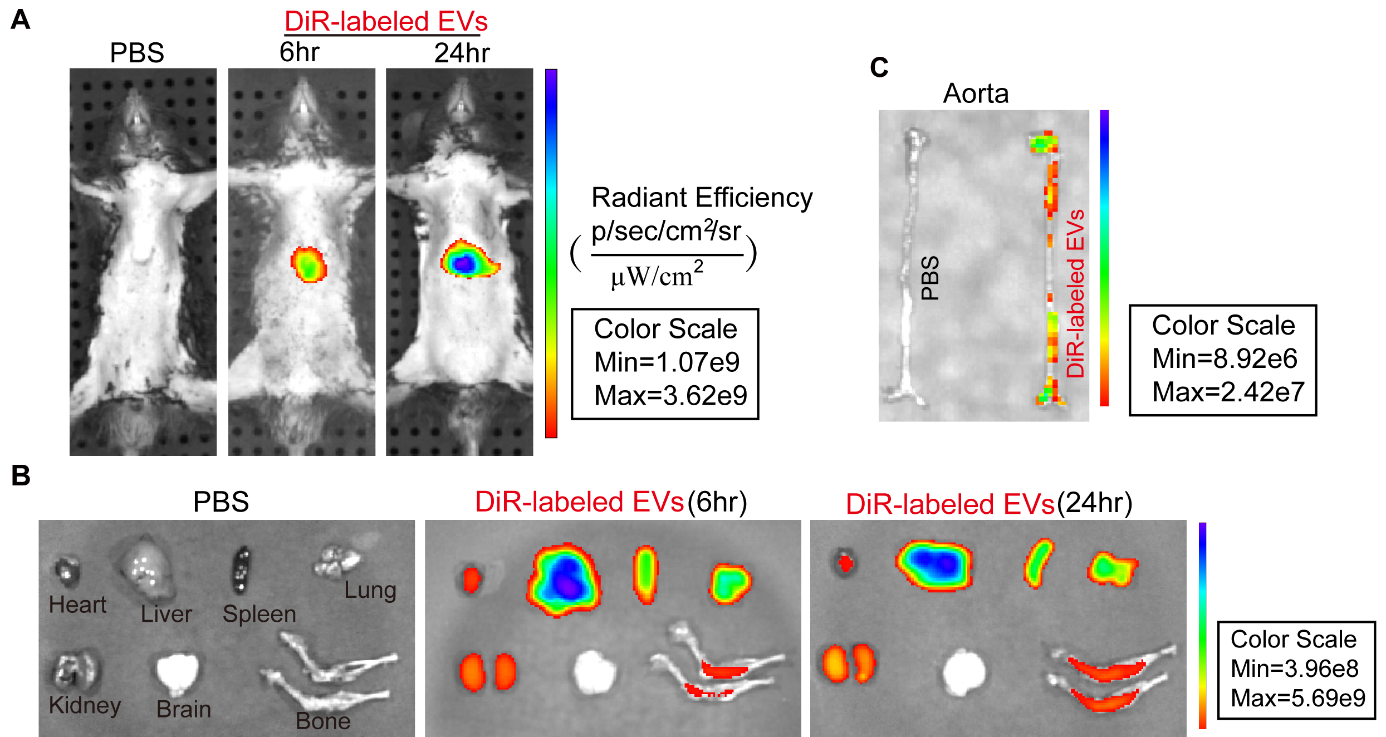


**Figure S5. SHep-EVs *in vivo* tracing analysis.**

**A.** In vivo imaging at 6 and 24 hours after tail vein injection of 75 μg DiR-labelled SHep-EVs or solvent control (i.e. SHep-EVs incubated with PBS instead of DiR) in 8-week-old C57 mice (n=3); **B.** Fluorescence imaging of organs at corresponding time points (n=3); **C.** Aortic fluorescence imaging at 24 hours after tail vein injection of 75 μg DiR-labelled SHep-EVs or solvent control (n=3).


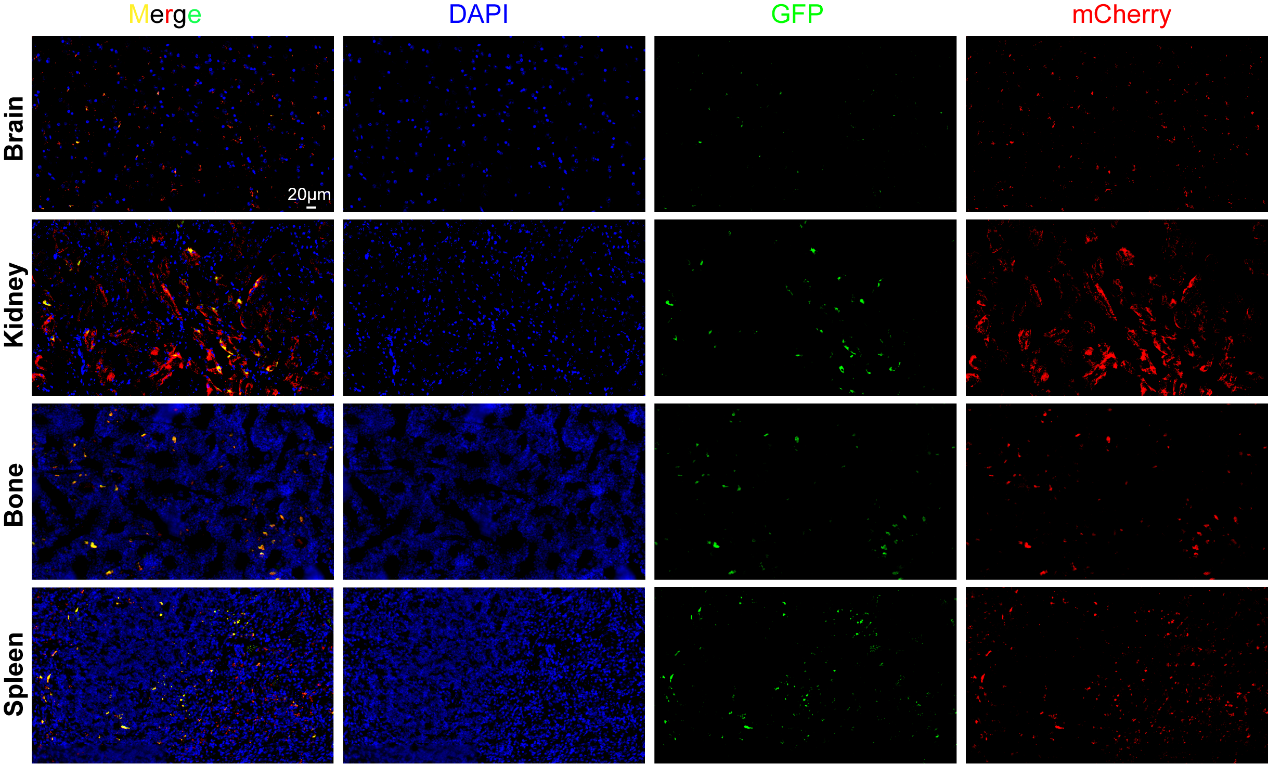


**Figure S6. Detection of Hep-EVs tissue distribution in hepatocyte-derived EVs tracer mice.**

Fluorescent signals analysis of eGFP (green) and mCherry (red) in the Brain, Kidney, Bone, and Spleen with EVs distributed in dots around the cell nucleus (in the cytoplasm), scale bar=20 μm.


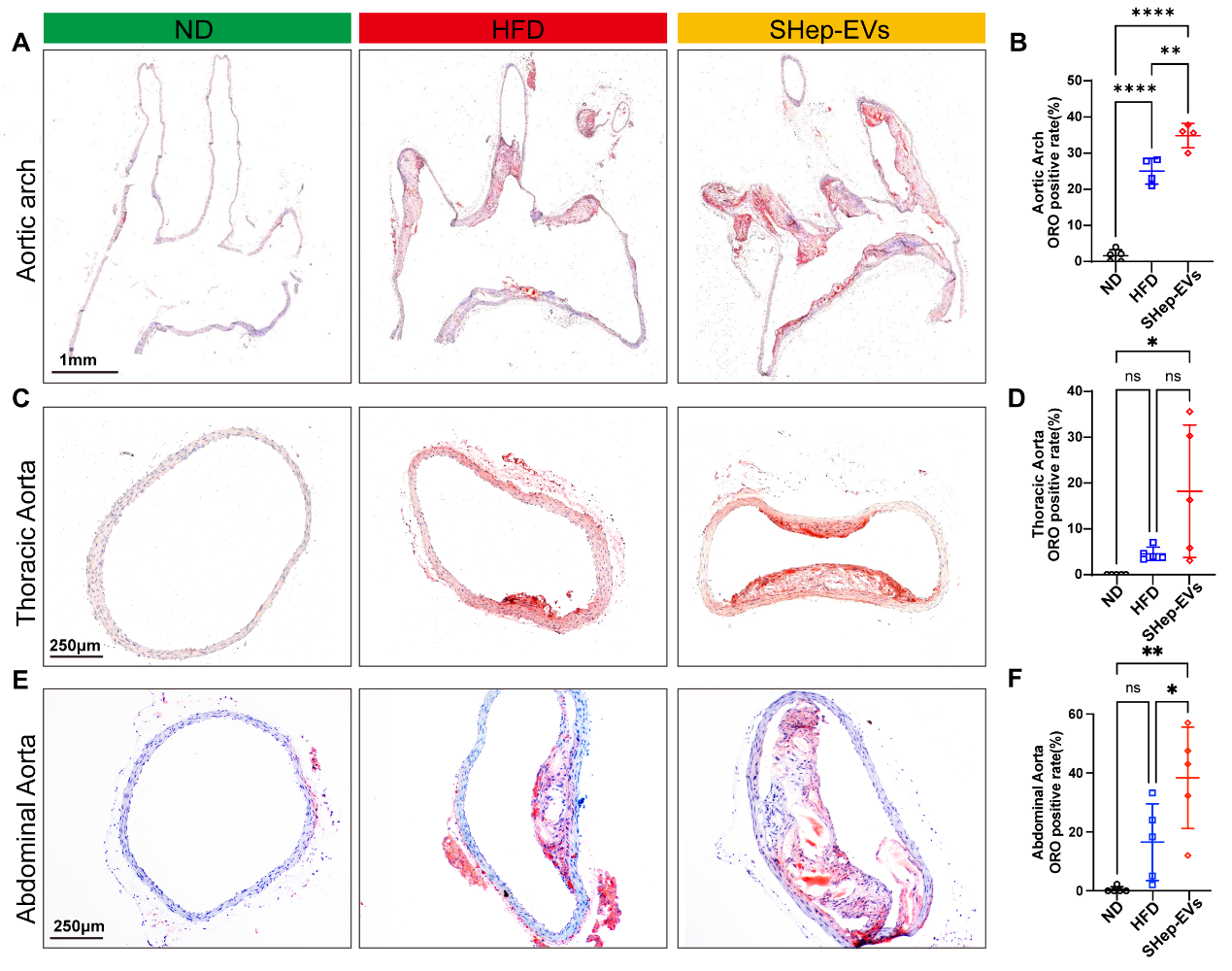


**Figure S7. Effects of SHep-EVs on atherosclerotic plaques in different parts of the aorta.**

**A, B.** ORO staining of the aortic arch and the statistical analysis of atherosclerotic lesions (n=4 or 5), scale bar=1 mm; **C, D.** Thoracic aorta ORO staining and the statistical analysis (n=5), scale bar=250 μm; **E, F.** Abdominal aorta ORO staining and the statistical analysis (n= 5), scale bar=250 μm; Data are presented as mean±SD, *p* values were determined by one-way ANOVA followed by Tukey’s test, “*ns”* no significant difference, **p*< 0.05, ***p*< 0.01, *****p*< 0.0001.


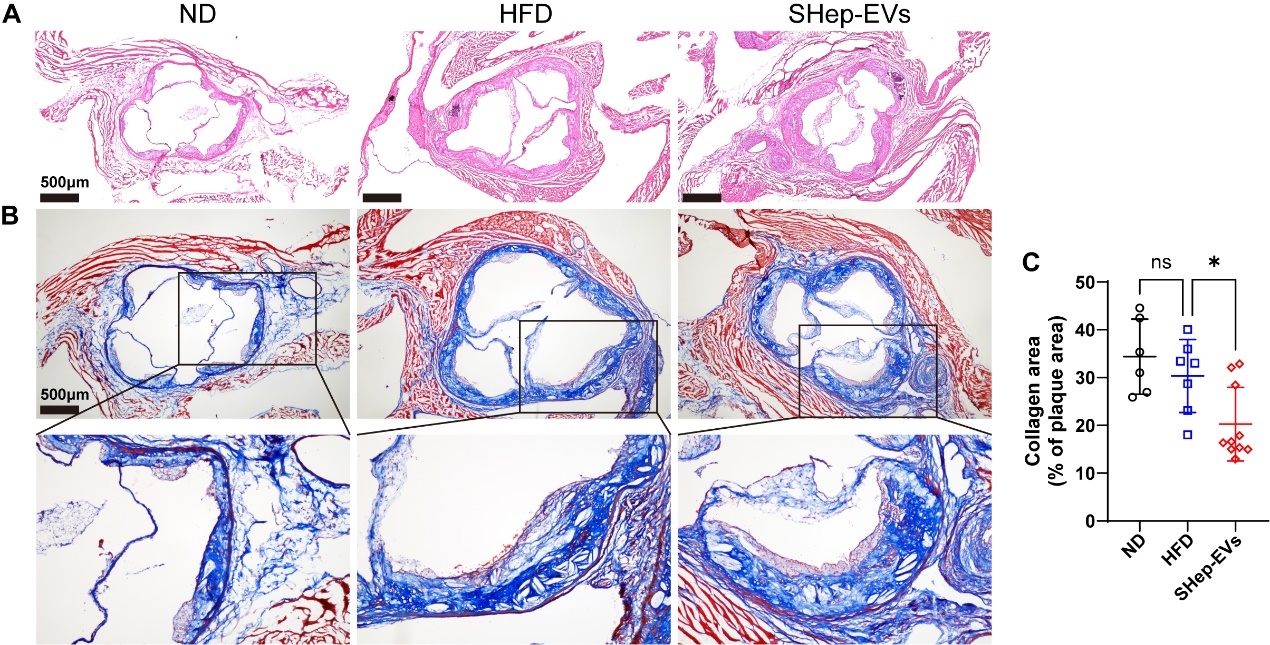


**Figure S8. SHep-EVs reduce atherosclerotic plaque collagen content.**

**A.** H&E staining of aortic sinus, scale bar=500 μm; **B-C.** Masson staining of aortic sinus and collagen area statistics (n=6-10), scale bar =500 μm. Data are presented as mean±SD, *p* values were determined by one-way ANOVA followed by Tukey’s test, “ns” no significant difference, **p*< 0.05.


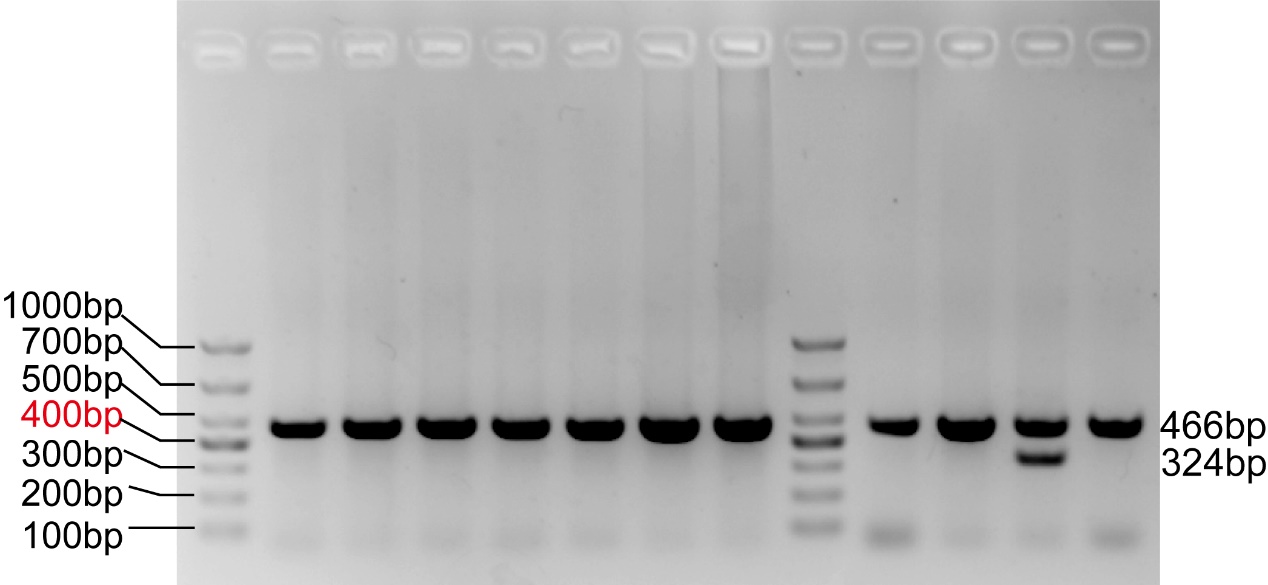


**Figure S9. Rab27^flox/flox^ mouse genotyping.**

Expected PCR Product: Flox:466bp, WT:324bp; double-banded: heterozygous.


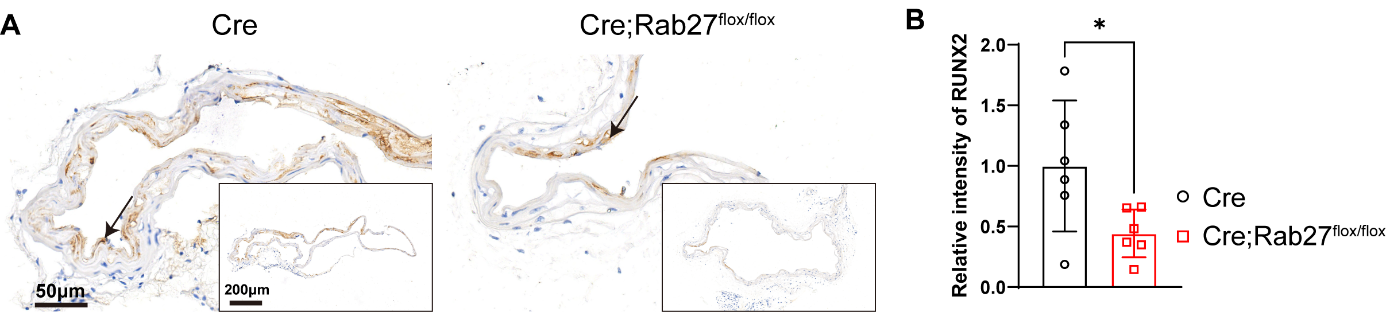


**Figure S10.** **Inhibition of SHep-EV secretion reduces the expression of the osteogenic marker RUNX2 in the aorta of MAFLD mice.**

Immunohistochemical staining of RUNX2 in the aorta and statistical analysis, scale bar=200 or 50μm; Data are presented as mean±SD, *p* values were determined by unpaired two-tailed Student’s t-test, ***p*＜0.01.


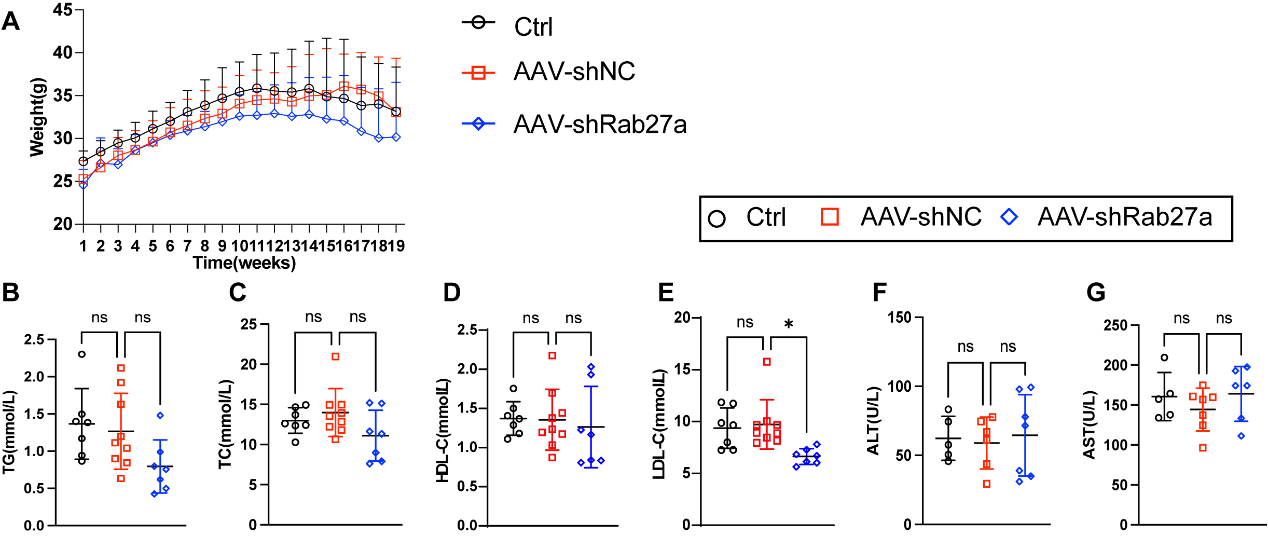


**Figure S11. Effects of liver-specific knockdown of Rab27a in ApoE^-/-^ mice on body weight, liver function, and blood lipids.**

**A.** Mouse weight; **B-E.** Blood lipid; **F, G.** Liver function; Data are presented as mean±SD, *p* values were determined by one-way ANOVA followed by Tukey’s test. "ns" no significant difference, "*" *p* < 0.05.


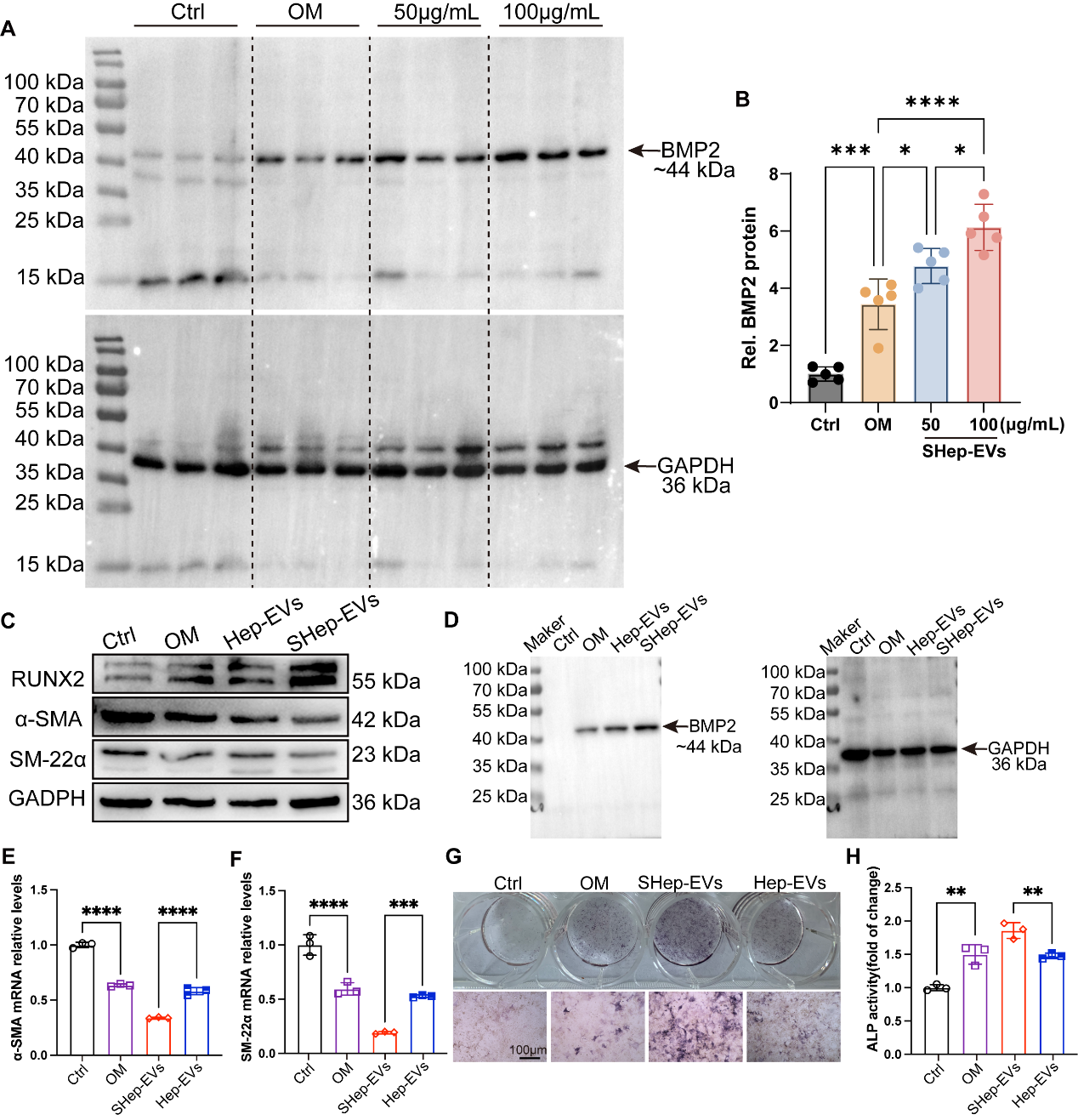


**Figure S12. Comparison of SHep-EVs and Hep-EVs on the osteogenic differentiation of VSMC.**

**A, B.** Western blot detection and statistical analysis of the expression of osteogenic marker BMP2 in VSMC treated by different concentrations of SHep-EVs for 7days (n=5); **C, D.** The expression of osteogenic marker RUNX2, BMP2 and smooth muscle contraction phenotypic markers α-SMA, SM-22α protein in VSMC treated by SHep-EVs or Hep-EVs for 7 days are detected by Western blot; **E, F** The expression of smooth muscle contraction phenotypic markers α-SMA, SM-22α mRNA in VSMC treated by SHep-EVs or Hep-EVs for 7 days are detected by qPCR(n=3); **G, H.** The ALP staining and activity analysis be applied to detect the osteogenic differentiation level in VSMC treated by SHep-EVs or Hep-EVs for 7 days(n=3), scale bar =100 μm. Data are presented as mean±SD, *p* values were determined by one-way ANOVA followed by Tukey’s test, “ns” no significant difference, **p*< 0.05, ***p*< 0.01, ****p*< 0.001, *****p*< 0.0001.


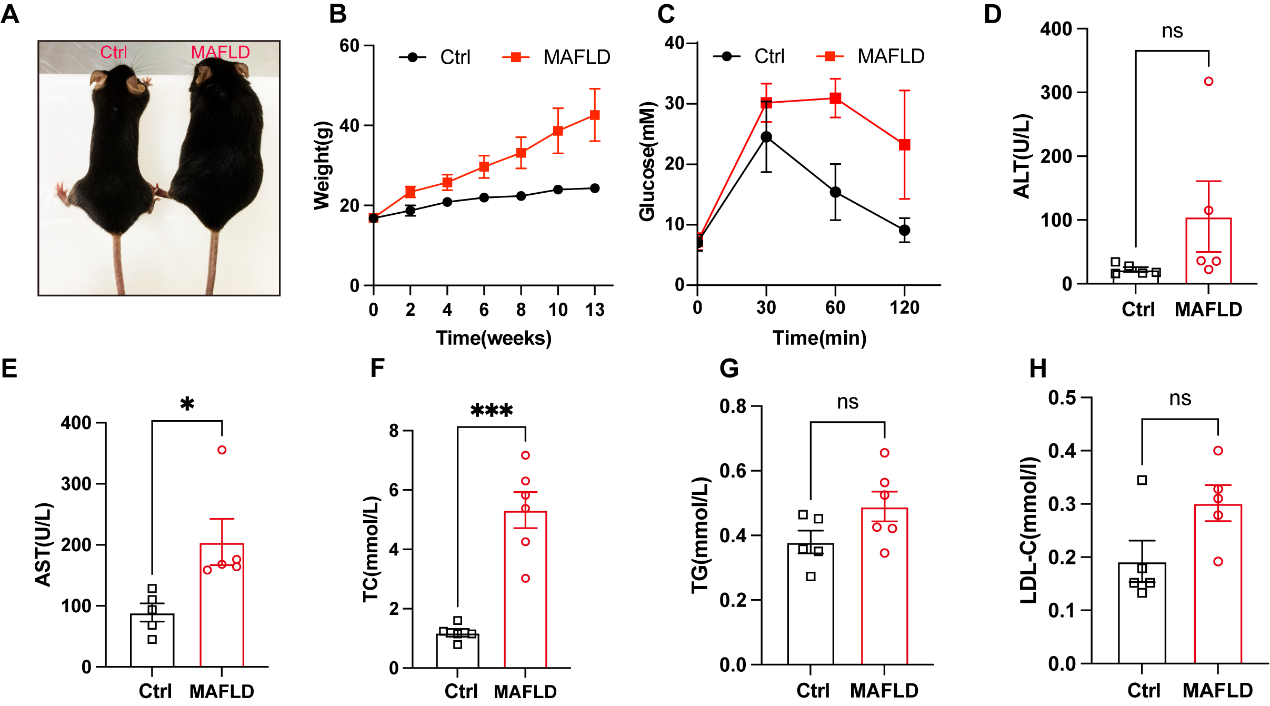


**Figure S13. General indicators of primary hepatocyte donor mice.**

**A.** Mouse body size comparison. **B.** Body weight comparison(n=10); **C.** Glucose tolerance test (n=10). **D, E.** Liver function analysis(n=5); **F-H.** Blood lipid comparison (n=5 or 6). Data are presented as mean±SD, *p* values were determined by unpaired two-tailed Student’s t-test, "ns" no significant difference, "*" *p* < 0.05, and "***" *p* < 0.001.


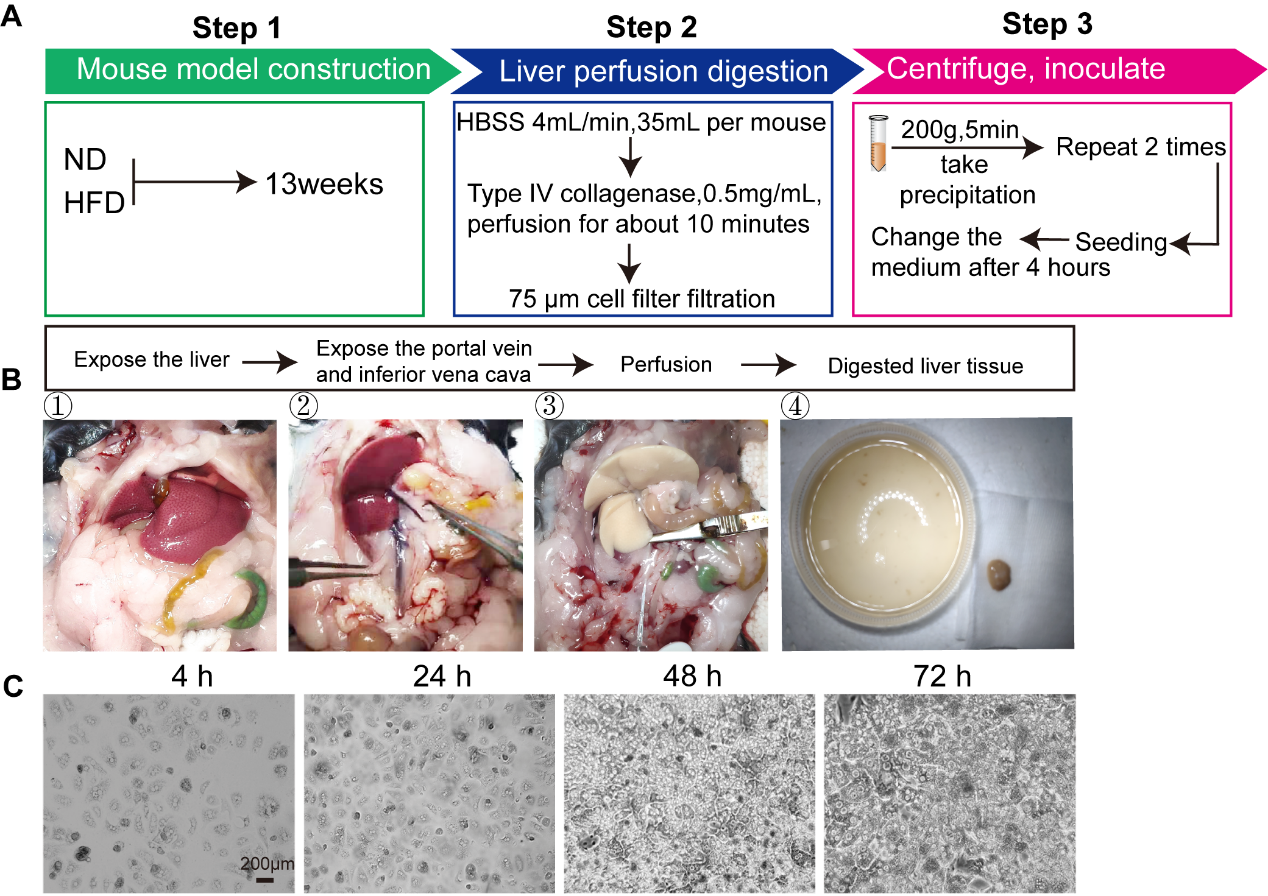


**Figure S14. Extraction and culture of primary mouse hepatocytes.**

**A.** Flow chart of primary mouse hepatocyte extraction, the detail process is shown in the experimental methods section; **B.** Representative images of the main steps of primary mouse hepatocyte extraction, ①exposing the liver, ②exposing the inferior vena cava and the portal vein, ③perfusion of HBSS or collagenase IV through the inferior vena cava, the liver was rapidly enlarged and whitened after the perfusion was successfully performed, and ④the perfused liver was dissociated into a cellular suspension, with a small amount of connective tissue remaining; **C.** Images of primary hepatocytes at different time points (4-72 h) after inoculation, scale bar =200 μm.


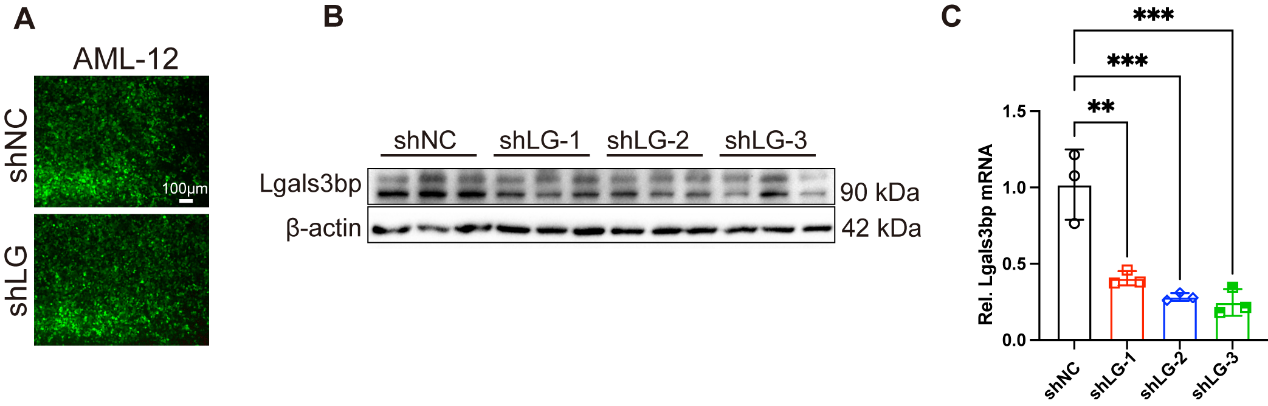


**Figure S15. Lgals3bp adenovirus transfection and knockdown efficiency analysis.**

**A.** AML-12 hepatocytes expressing strong eGFP signals after transfection of shRNA, scale bar=100 μm; **B.** Western bot used to detect the knockdown efficiency of Lgals3bp (n=3); **C.** qPCR used to detect the knockdown efficiency of Lgals3bp (n=3). Data are presented as mean±SD, and *p* values were determined by one-way ANOVA followed by Tukey’s test, "**" *p* < 0.01, and "***" *p* < 0.001.


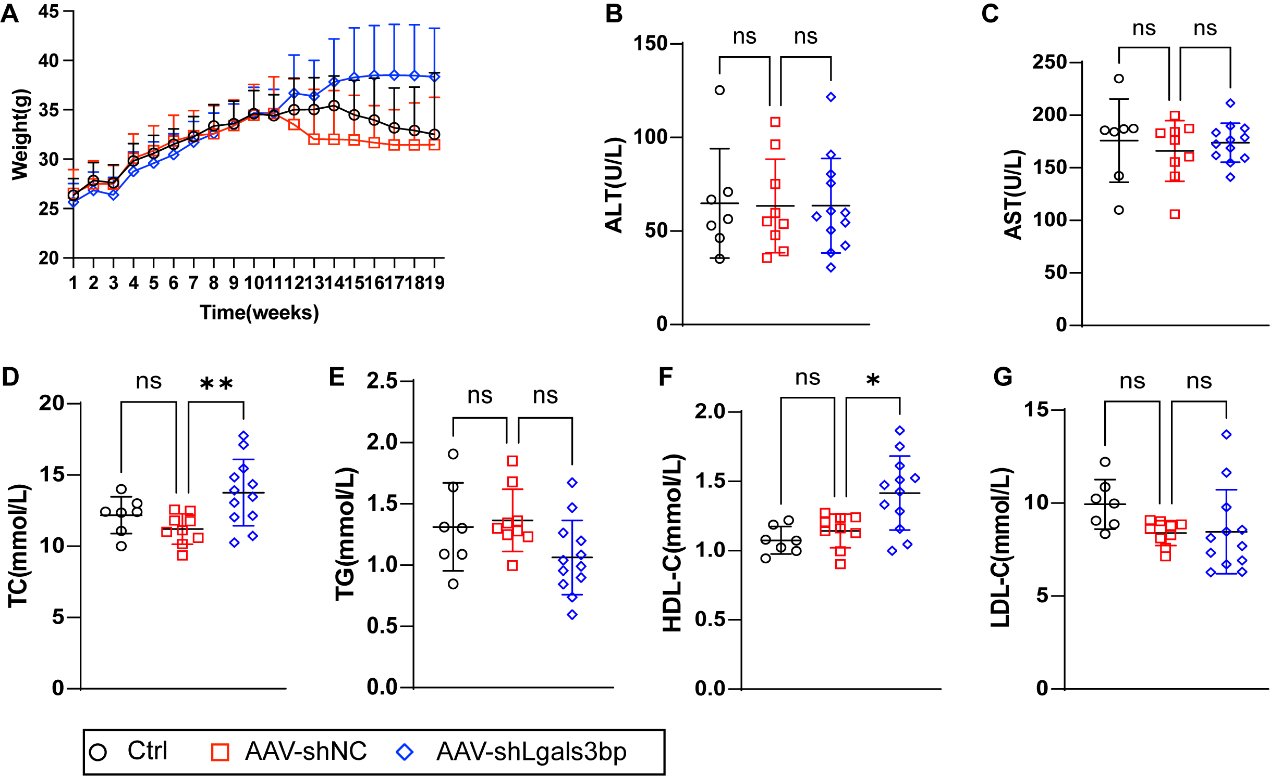


**Figure S16. Effects of liver-specific knockdown of Lgals3bp in ApoE^-/-^ mice on body weight, liver function, and blood lipids.**

**A.** Mouse weight; **B, C.** Liver function; **D-G.** Blood lipid. Data are presented as mean±SD, and *p* values were determined by one-way ANOVA followed by Tukey’s test. "ns" no significant difference, "*" *p* < 0.05, "**" *p* < 0.01.
